# A non‐redundant role of EAAT3 for ATP synthesis mediated by GDH in dopaminergic neuronal cells: a new avenue for glutamate metabolism and protection in Parkinson's disease

**DOI:** 10.1111/febs.70053

**Published:** 2025-03-05

**Authors:** Alessandra Preziuso, Tiziano Serfilippi, Marwa Toujani, Valentina Terenzi, Salvatore Amoroso, Simona Magi, Vincenzo Lariccia, Silvia Piccirillo

**Affiliations:** ^1^ Department of Biomedical Sciences and Public Health School of Medicine, University “Politecnica delle Marche” Ancona Italy; ^2^ Department of Pediatrics Meyer Children's University Hospital Florence Italy

**Keywords:** energy deficit, mitochondria, neuroprotection, oxidative stress, Parkinson's disease

## Abstract

Parkinson's disease (PD) is a devastating neurodegenerative disorder with a distinct loss of the nigrostriatal dopaminergic pathway. Despite the multiplicity in etiology, alterations that disrupt neuronal integrity can be traced back to defects in fundamental processes that typically run under mitochondrial inputs. Evidence indicates that mitochondrial activities are hierarchically integrated with the energetic performance of these organelles, so that an interesting perspective holds that interventions aimed at improving mitochondrial bioenergetics can potentially mitigate the severity of PD phenotype expression. In this mechanistic framework, approaches that facilitate the mitochondrial anaplerotic use of glutamate (Glut) might counteract the detrimental shift from Glut metabolism, which is typically altered in PD, to excessive Glut transmission that feeds excitotoxicity and the neurodegenerative spiral. In this study, we investigated whether the enhancement of glutamate dehydrogenase (GDH) activity, by using the GDH activator 2‐aminobicyclo‐(2,2,1)‐heptane‐2‐carboxylic acid (BCH), has neuroprotective potential against PD injury. In both retinoic acid‐differentiated SH‐SY5Y cells and primary rat mesencephalic neurons challenged with α‐synuclein plus rotenone to mimic PD, BCH‐dependent GDH activation significantly ameliorated cell viability, improved mitochondrial ATP synthesis and lessened to control levels the cellular redox burden. Strikingly, we collected evidence for the existence of a functional axis connecting GDH activity to a specific intracellular pool of the Excitatory Amino Acid Transporters (EAATs), namely the EAAT3. Overall, our results reveal a novel and non‐redundant role of EAAT3 for GDH‐dependent protection against PD injury, which may inspire new pharmacological approaches against PD pathology.

## Graphical abstract

Parkinson's disease (PD) is a severe brain disorder caused by the loss of dopaminergic neurons. This study found that activating the enzyme glutamate dehydrogenase (GDH), by using the GDH activator BCH, protects cells and improves their energy production. It also highlights the previously undescribed role of EAAT3 in GDH‐dependent protection against PD injury, suggesting new potential treatments for the disease.

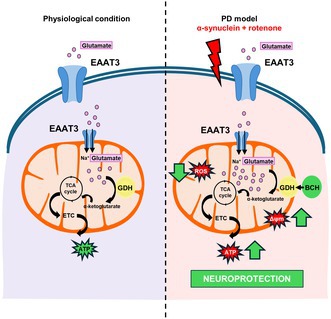

**Abbreviations**
2‐DG: 2‐deoxyglucose
AD: Alzheimer's disease
BCH: 2‐aminobicyclo‐(2,2,1)‐heptane‐2‐carboxylic acid
Ca^2+^
: calcium
EAATs: excitatory amino acid transporters
FCCP: carbonyl cyanide p‐trifluoromethoxyphenylhydrazone
GDH: glutamate dehydrogenase enzyme
LDH: lactate dehydrogenase
PD: Parkinson's disease
RNAi: RNA interference
ROS: reactive oxygen species
Rot: Rotenone
siEAAT3: siRNA against EAAT3
SNpc: substantia nigra pars compacta
TCA: tricarboxylic acid cycle
TFB‐TBOA: (3S)‐3‐[[3‐[[4‐(Trifluoromethyl)benzoyl] amino]phenyl]methoxy]‐L‐aspartic acid
TMRE: tetramethylrhodamine ethylester
α‐syn: α‐synuclein
ΔΨ_m_
: mitochondrial membrane potential

## Introduction

Parkinson's disease (PD) is a devastating neurodegenerative disorder associated to a distinct degeneration of the nigrostriatal dopaminergic pathway [1]. Despite the multiplicity in etiology and heterogeneity in the phenotype expression, disruption in brain cell metabolism is a common outcome of both genetic and environmental factors [2]. Indeed, an interesting perspective holds that metabolic alterations drive and worsen the progression of PD in a feedforward spiral of decline [3, 4]. Signs of reduced metabolic efficiency emerge since the pre‐symptomatic stages of PD, which may progressively disable nigrostriatal synergy, as well as compromise signaling and computational abilities of basal ganglia nuclei. Moreover, glutamatergic dysfunctions [5, 6, 7] have pathological relevance for the amplification of damage in the substantia nigra pars compacta (SNpc) and the appearance of PD motor symptoms [8, 9, 10]. The exact mechanism behind development of PD is still puzzling. However, there is enough ground for the pathological contribution of α‐synuclein (α‐syn) accumulation and exposure to environmental toxins, including rotenone (rot), resulting in the loss of dopaminergic neurons in the SNpc [11, 12, 13, 14, 15]. Importantly, recombinant α‐syn oligomers have been observed to transiently and dynamically interact with mitochondria. This interaction leads to the reduction of mitochondrial membrane potential, intracellular ATP production, mitochondrial fragmentation, and degradation along with a decrease in mitochondrial content [16, 17]. All the above‐mentioned alterations can be traced back to defects in fundamental processes that typically run under mitochondrial inputs, such as energy production, calcium (Ca^2+^) homeostasis, intracellular redox balance, and maintenance of inner membrane potential. On this background, an effective control on mitochondrial bioenergetics may have major ramifications for the resilience of post‐mitotic brain cells against PD injury. The rationale of this strategy is also supported by the comprehensive concept that connects neuronal energy metabolism to the control of aging and longevity of the human brain [18]. In this connection, a recent study provided evidence that a combination of succinate salt of choline and nicotinamide can safeguard astrocytes and neurons from excitotoxic damage and protect neurons with familial forms of PD from neurodegeneration [19]. Using a complementary approach, we previously demonstrated that the stimulation of energy metabolism, through the provision of glutamate (Glut) as alternative metabolic source, ameliorated cell viability [20]. This was achieved by promoting the intracellular ATP synthesis and preserving the overall mitochondrial functions in an *in vitro* model of PD based on retinoic acid (RA)‐differentiated SH‐SY5Y neuroblastoma dopaminergic cells exposed to α‐syn plus rot treatment [20]. Of particular interest is the observation that the pro‐survival effects of Glut against α‐syn plus rot‐induced neurotoxicity depend on the activity of Excitatory Amino Acid Transporters 3 (EAAT3) [20]. EAATs are crucial for regulating Glut levels by facilitating its uptake from the extracellular space. This process is essential for sustaining proper glutamatergic signaling and preventing the harmful accumulation of Glut, which could otherwise lead to excitotoxicity [21]. In line with this concept, research into the deregulation of Glut metabolism in neurodegenerative disorders has identified mitochondrial glutamate dehydrogenase (GDH) as a promising therapeutic target [22]. GDH is a key enzyme that controls anaplerotic flux fulfilling the tricarboxylic acid (TCA) cycle. It converts Glut into α‐ketoglutarate, thereby influencing oxidative balance and bioenergetic functions of mitochondria [23]. Indeed, high levels of GDH and EAAT3 expression have been observed in mesencephalic dopaminergic neurons, where the inhibition of GDH expression has been shown to be particularly toxic, especially during the early stages of this inhibition [24]. Moreover, early evidence documented GDH‐deficient activity in patients with various types of neurodegenerative disorders [25, 26, 27]. Of note, enhancing the expression and function of EAATs has been found to mitigate the loss of dopaminergic neurons in the striatum and substantia nigra in models of PD [28]. In this connection, the activation of mitochondrial GDH, by using the leucine analogue 2‐aminobicyclo‐(2,2,1)‐heptane‐2‐carboxylic acid (BCH), has been demonstrated to protect neurons against metabolic failure and excitotoxicity in both *in vivo* mouse model of middle artery occlusion and *in vitro* oxygen/glucose depletion model [29].

Considering the crucial role of mitochondrial activity in controlling energy provision and cellular homeostasis, in the present study we explored whether the activation of GDH is a feasible approach to stimulate mitochondrial bioenergetics and survival in *in vitro* models of PD. To test this hypothesis, we used both RA‐differentiated SH‐SY5Y neuroblastoma cells and primary rat mesencephalic neurons exposed to α‐syn plus rot [20] to investigate whether stimulating Glut catabolism induced through the activation of GDH can enhance the mitochondrial bioenergetics and protect cells against energy failure.

## Results

### BCH‐dependent GDH activation promotes ATP synthesis, which specifically requires EAAT3 in RA‐differentiated SH‐SY5Y cells

**Fig. 1:**
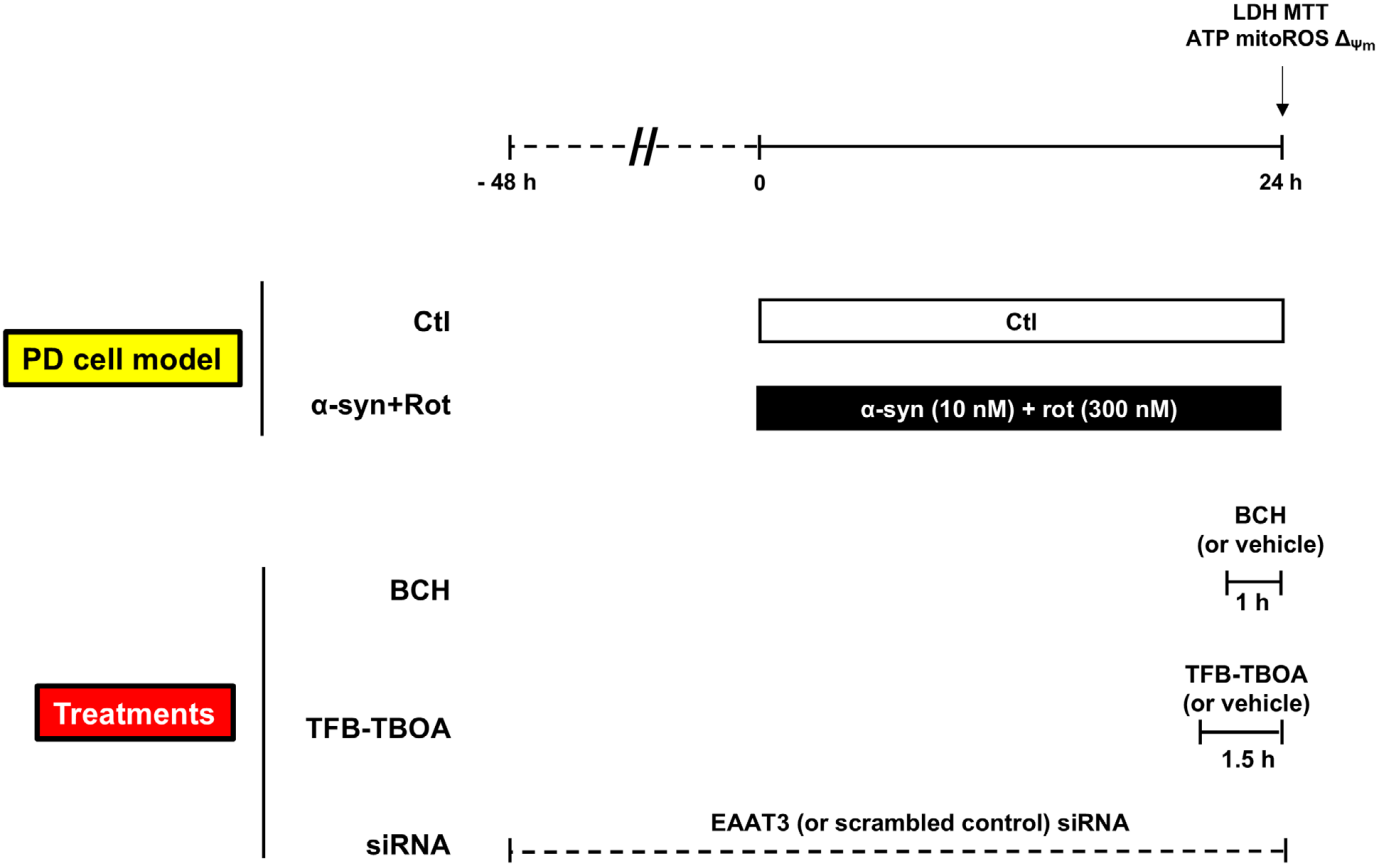
Timeline of the experimental protocol. For the PD cell model, RA‐differentiated SH‐SY5Y cells and primary rat mesencephalic neurons were treated with α‐syn (10 nm) and rot (300 nm) for 24 h. BCH (1 mm) and TFB‐TBOA (1 μm) were added during the last 1 h and the last 1.5 h of the experimental protocol, respectively. Silencing of EAAT3 was achieved by incubating cells with EAAT3 siRNA oligonucleotide (80 nm) for 48 h. Cell viability, ATP levels, ROS, and ΔΨ_m_ were evaluated at the end of the experimental protocol. α‐syn, α‐synuclein; BCH, 2‐aminobicyclo‐(2,2,1)‐heptane‐2‐carboxylic acid; Ctl, control; Rot, rotenone; siEAAT3, siRNA against EAAT3.

**Fig. 2:**
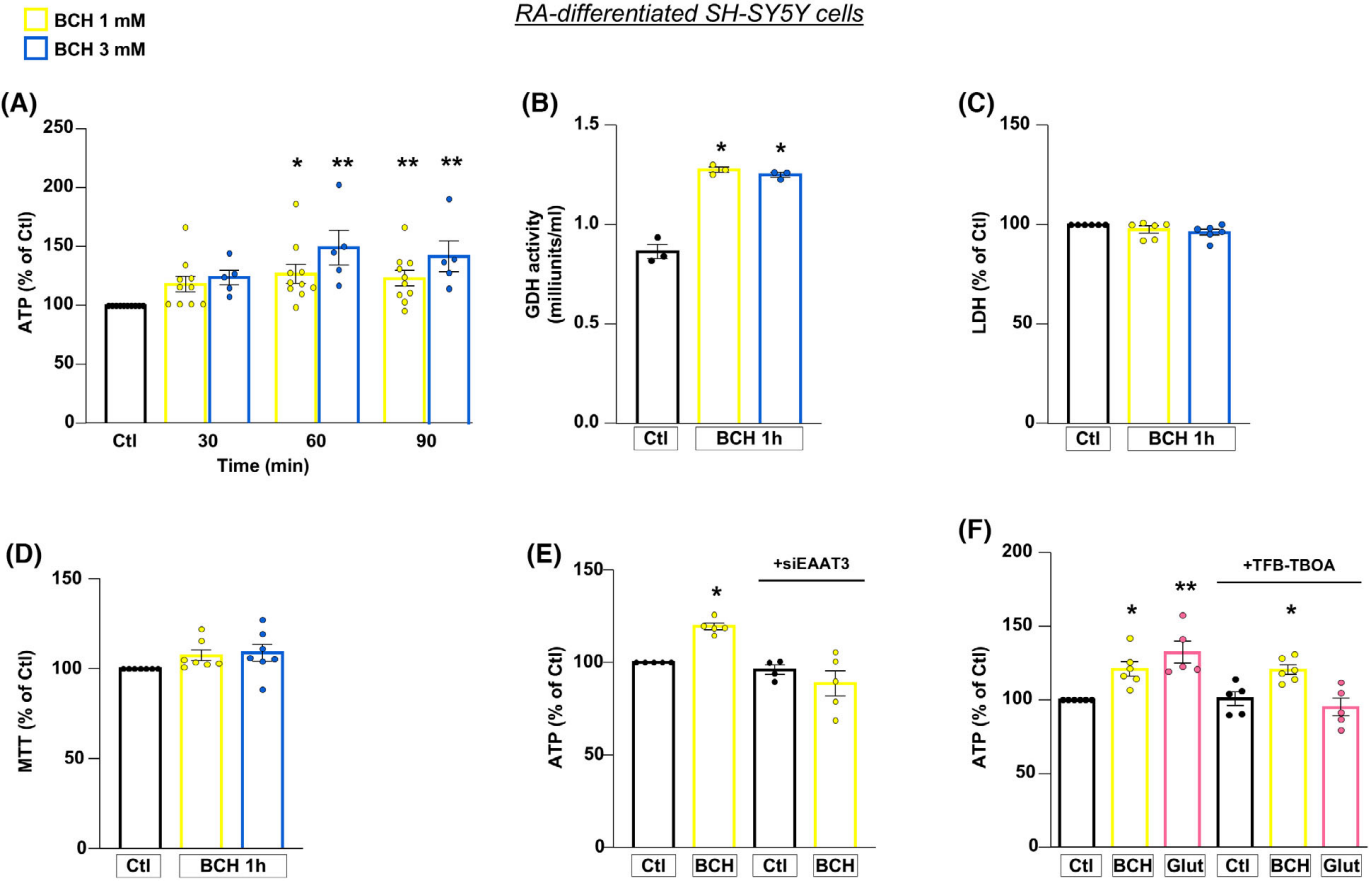
BCH‐dependent GDH activation promotes ATP synthesis in RA‐differentiated SH‐SY5Y cells: essential role of EAAT3 glutamate transporter. (A) ATP level was evaluated after 30, 60, 90 min exposure to BCH (1 or 3 mm). (B) GDH activity and (C, D) cell viability, measured by means of extracellular LDH activity (C) and MTT assay (D), were evaluated after 1 h exposure to BCH (1 or 3 mm). (E) After knocking down EAAT3 expression, intracellular ATP content was measured after 1 h exposure to BCH (1 mm). (F) Evaluation of intracellular ATP content after 30 min exposure to TFB‐TBOA (1 μm) followed by 1 h of BCH (1 mm) or Glut (500 μm). Statistical differences among means were evaluated by one‐way ANOVA followed by Dunnett's *post hoc* test. Data are represented as means ± SEM. (A) *Significant vs Ctl (*P* < 0.01); **significant vs Ctl (*P* < 0.05). For 1 mm BCH *n* = 10, for 3 mm BCH *n* = 5 independent experiments; (B) *Significant vs Ctl (*P* < 0.0001). *n* = 3 independent experiments; (C) *n* = 6 independent experiments; (D) *n* = 7 independent experiments; (E) *Significant vs all groups (*P* < 0.01 vs Ctl and siEAAT3; *P* < 0.001 vs siEAAT3 + BCH). *n* = at least 4 independent experiments; (F) *Significant vs Ctl and TFB‐TBOA (*P* < 0.05), and vs Glut + TFB‐TBOA (*P* < 0.01); **significant vs Ctl and TFB‐TBOA (*P* < 0.001), and vs Glut + TFB‐TBOA (*P* < 0.0001). *n* = at least 3 independent experiments. Glut, glutamate.

To explore the role of GDH in promoting the enhancement of energy metabolism in an *in vitro* model of PD based on α‐syn plus rot treatment [20], we used BCH as a GDH activator in both RA‐differentiated SH‐SY5Y cells and primary rat mesencephalic neurons, as reported in the timeline of the experimental protocol (Fig. 1) [29, 30]. We initially analyzed the effect of BCH in physiological setting by exposing cells to 1 and 3 mm BCH concentrations for 30, 60, and 90 min. As shown in Fig. 2A, BCH treatment significantly increased the intracellular ATP level at both 1 and 3 mm after 60 and 90 min (min) in RA‐differentiated SH‐SY5Y cells (Fig. 2A). BCH also stimulated the enhancement of GDH activity at both concentrations tested in the mitochondrial fraction from RA‐differentiated SH‐SY5Y cells (Fig. 2B) without displaying any sign of toxicity (Fig. 2C,D). Based on these results, we chose 1 mm and 1 h as BCH working concentration and time of exposure, respectively, for the following experiments. To examine the involvement of EAATs in the BCH‐dependent GDH activation, we used both pharmacological and genetic approaches. In particular, when cells were exposed to small interference RNA against EAAT3 (siEAAT3), the increase in ATP synthesis induced by BCH was completely abolished (Fig. 2E). On the contrary, the treatment with (3S)‐3‐[[3‐[[4‐(Trifluoromethyl)benzoyl] amino]phenyl]methoxy]‐L‐aspartic acid (TFB‐TBOA, 1 μm), which is a non‐transportable inhibitor expected to affect only EAATs located on plasma membrane [31], did not interfere with the stimulation of ATP synthesis induced by BCH (Fig. 2F). A cell surface confined block of TFB‐TBOA was also supported by a different set of experiments in which cells were exposed to Glut in the presence and/or in the absence of TFB‐TBOA (Fig. 2F). As expected, Glut induced a significant increase in ATP production which was completely counteracted by TFB‐TBOA, indicating that blockade of plasma membrane EAAT transporters can fully abolish responses to Glut but not to BCH (Fig. 2F). Both EAAT3 silencing and TFB‐TBOA exposure did not exert any cytotoxic effects (Fig. S1A–D). Overall, the ability of siEAAT3 (but not TFB‐TBOA) to fully prevent the rise of ATP synthesis induced by BCH supports the hypothesis that an intracellular pool of EAAT3 is specifically involved in the metabolic response of BCH.

### BCH‐dependent GDH activation prevents fall in ATP level and injury in RA‐differentiated SH‐SY5Y cells exposed to α‐syn plus rot

**Fig. 3:**
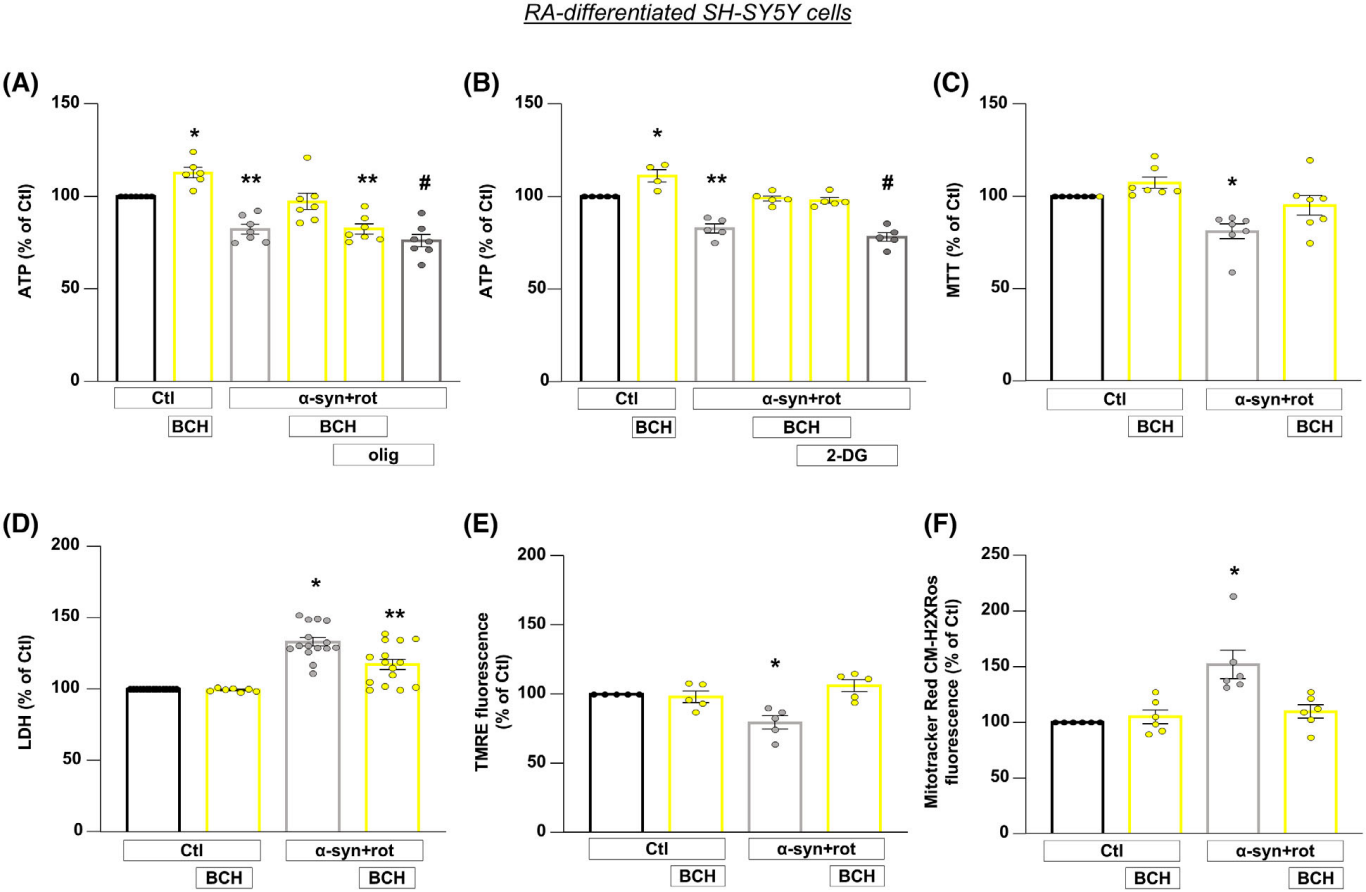
BCH‐dependent GDH activation prevents loss of mitochondrial ATP production and injury in RA‐differentiated SH‐SY5Y cells exposed to α‐syn plus rot. (A, B) The effect of BCH (1 mm) on intracellular ATP levels were assessed under physiological condition and in cells exposed to α‐syn plus rot (α‐syn + rot), with/without oligomycin (olig, 3 μg·mL^−1^) (A) or with/without 2‐deoxyglucose (2‐DG, 2 mm) (B). The effects of BCH (1 mm) on cell viability were assessed by MTT (C) and LDH assay (D), whereas mitochondrial membrane potential and mitochondrial ROS production were respectively assessed by monitoring TMRE (E) and MitoTracker Red CM‐H2XRos fluorescence intensity (F) in Ctl and α‐syn + rot treated cells. Statistical differences among means were evaluated by one‐way ANOVA followed by Dunnett's *post hoc* test. Data are represented as means ± SEM. (A) *Significant vs all groups (*P* < 0.05 vs Ctl, *P* < 0.0001 vs α‐syn + rot, α‐syn + rot + BCH + oligo and α‐syn + rot + oligo, *P* < 0.01 vs α‐syn + rot + BCH); **significant vs Ctl (*P* < 0.001), BCH (*P* < 0.0001) and α‐syn + rot + BCH (*P* < 0.01); #significant vs Ctl, BCH and α‐syn + rot + BCH (*P* < 0.0001). *n* = at least 6 independent experiments; (B) *Significant vs all groups (*P* < 0.01 vs Ctl and α‐syn + rot + BCH, *P* < 0.0001 vs α‐syn + rot and α‐syn + rot +2‐DG, *P* < 0.001 vs α‐syn + rot + BCH + 2‐DG); **significant vs control groups and α‐syn + rot + BCH (*P* < 0.0001), vs α‐syn + rot + BCH (*P* < 0.01) and vs α‐syn + rot + BCH + 2‐DG (*P* < 0.001); #significant vs control groups, α‐syn + rot + BCH and α‐syn + rot + BCH + 2‐DG (*P* < 0.0001). *n* = at least 4 independent experiments; (C) *Significant vs all groups (*P* < 0.01 vs Ctl, *P* < 0.001 vs BCH, *P* < 0.05 vs α‐syn + rot + BCH). *n* = 7 independent experiments; (D) *Significant vs all groups (*P* < 0.0001 vs Ctl and BCH, *P* < 0.001 vs α‐syn + rot + BCH); **Significant vs all groups (*P* > 0.0001 vs Ctl), vs BCH and α‐syn + rot + BCH (*P* < 0.001). *n* = at least 7 independent experiments; (E) *Significant vs all groups (*P* < 0.01 vs control groups; *P* < 0.001 vs α‐syn + rot + BCH). *n* = 5 independent experiments; (F) *Significant vs all groups (*P* < 0.001 vs control groups; *P* < 0.01 vs α‐syn + rot + BCH). *n* = 6 independent experiments.

To investigate whether BCH‐dependent GDH activation could exert protective role in PD model, we exposed RA‐differentiated SH‐SY5Y cells to α‐syn plus rot treatment (Fig. 1). As previously reported, when cells were exposed to α‐syn plus rot both ATP synthesis and cell viability are significantly compromised [20]. As shown in Fig. 3, BCH rescued the drop of ATP level (Fig. 3A,B) and protected cells against α‐syn plus rot toxicity (Fig. 3C,D). To analyze the bioenergetic route by which BCH stimulates the synthesis of ATP level, we exposed cells to oligomycin (3 μg·mL^−1^), an ATP synthase inhibitor, and to 2‐deoxyglucose (2‐DG, 2 mm), an inhibitor of glycolysis [32, 33, 34, 35, 36, 37] (Fig. 3A,B). The exposure to oligomycin prevented the rise of ATP level (Fig. 3A), while 2‐DG did not prevent the increase in ATP content induced by BCH (Fig. 3B). Of note, even when 2‐DG was tested at higher concentration (10 mm), BCH was still able to stimulate ATP production to a similar extent as observed with BCH alone (Fig. S2). Therefore, we reasoned that TCA cycle and oxidative phosphorylation are the way used by BCH to promote the ATP synthesis through the enhancement of Glut oxidation by GDH. The drop of ATP synthesis correlates with the loss of mitochondrial membrane potential (ΔΨ_m_); therefore, we analyzed the effect of α‐syn plus rot challenge and BCH on ΔΨ_m_. The treatment with α‐syn plus rot caused the collapse of ΔΨ_m_ which was prevented by BCH (Fig. 3E). We observed that BCH also reduced the increase in mitochondrial reactive oxygen species (ROS) production induced by α‐syn plus rot treatment (Fig. 3F), supporting the ability of the GDH activator to protect cells from α‐syn plus rot damage.

### Essential role of intracellular EAAT3 for the BCH‐mediated neuroprotection against α‐syn plus rot neurotoxicity in RA‐differentiated SH‐SY5Y cells

**Fig. 4:**
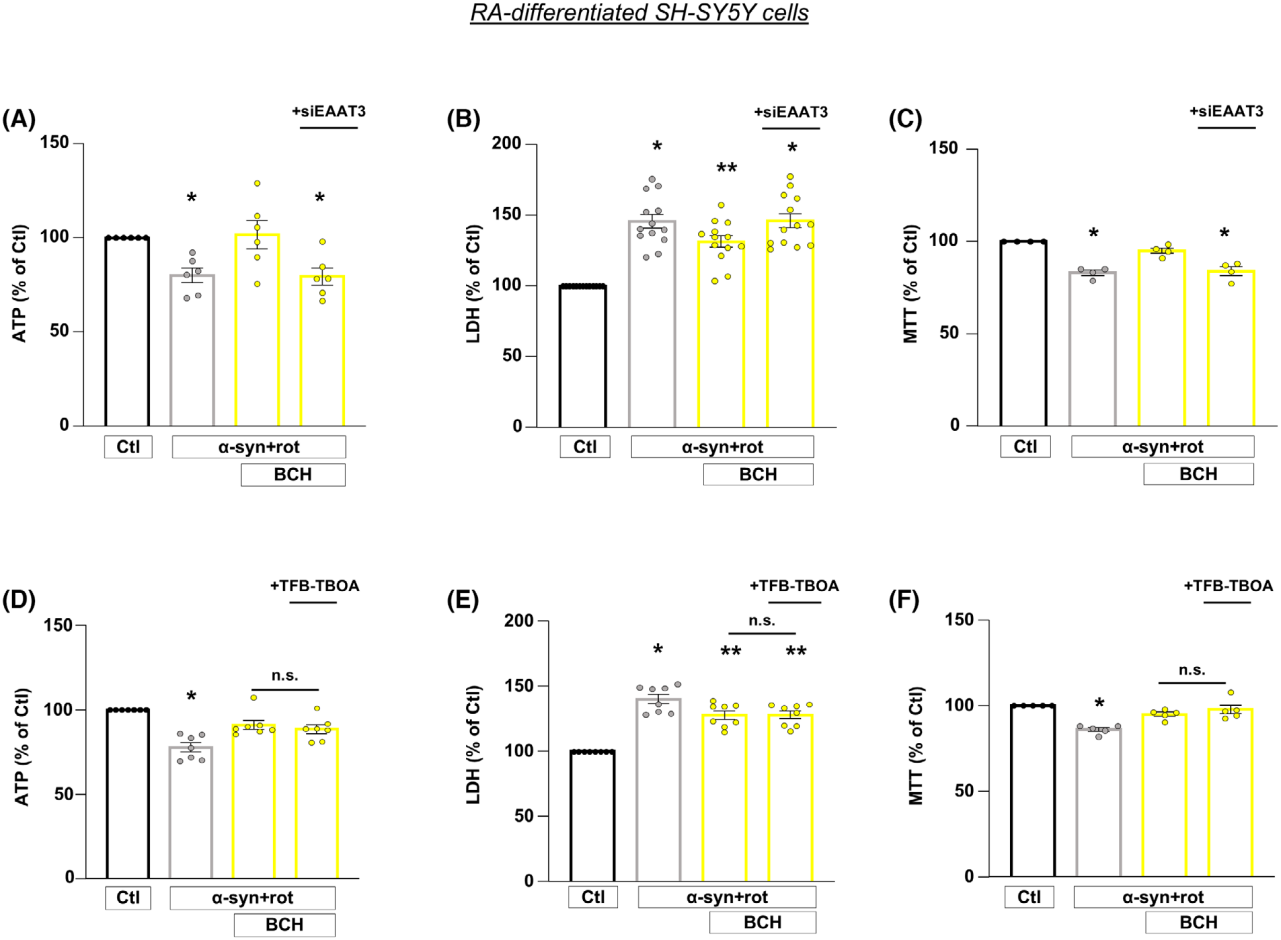
Involvement of EAAT3 glutamate transporters in the neuroprotective effects of BCH against α‐syn plus rot neurotoxicity in RA‐differentiated SH‐SY5Y cells. (A–C) The effects of BCH on intracellular ATP levels (A) and cell viability (B, C) were assessed in cells exposed to α‐syn plus rot after 48 h silencing of EAAT3 expression. (D–F) The effects of TFB‐TBOA (1 μm) on intracellular ATP levels (D) and cell viability (E, F) were assessed in cells exposed to α‐syn plus rot. Statistical differences among means were evaluated by one‐way ANOVA followed by Dunnett's *post hoc* test. Data are represented as means ± SEM. (A) *Significant vs Ctl and α‐syn + rot + BCH (*P* < 0.05). *n* = 6 independent experiments; (B) *Significant vs Ctl (*P* < 0.0001) and α‐syn + rot + BCH (*P* < 0.05); **significant vs all groups (*P* < 0.0001 vs Ctl, *P* < 0.05 vs α‐syn + rot + BCH and α‐syn + rot + BCH + siEAAT3). *n* = 13 independent experiments; (C) *Significant vs Ctl (*P* < 0.0001) and α‐syn + rot + BCH (*P* < 0.001). *n* = 4 independent experiments; (D) *Significant vs Ctl (*P* < 0.0001), α‐syn + rot + BCH (*P* < 0.01) and α‐syn + rot + BCH + TFB‐TBOA (*P* < 0.05). *n* = 7 independent experiments; (E) *Significant vs Ctl (*P* < 0.0001), α‐syn + rot + BCH and α‐syn + rot + BCH + TFB‐TBOA (*P* < 0.05); **significant vs Ctl (*P* < 0.0001) and α‐syn + rot (*P* < 0.05). *n* = 8 independent experiments; (F) *Significant vs Ctl (*P* < 0.0001), α‐syn + rot + BCH (*P* < 0.01) and α‐syn + rot + BCH + TFB‐TBOA (*P* < 0.001). *n* = 5 independent experiments.

Considering that BCH stimulates GDH activity and that EAATs transporters are distributed at both cell surface and intracellular (mitochondrial) levels [21, 38, 39], we evaluated the relative contribution of these EAAT pools to the protective effects induced by BCH by using RNA interference (RNAi) and pharmacological approaches described above. Firstly, siRNA against EAAT3 was performed on RA‐differentiated SH‐SY5Y cells. Silencing of EAAT3 completely prevented the neuroprotective effects of BCH in terms of ATP synthesis and cell viability (Fig. 4A–C), revealing that the EAAT3 transporter is specifically involved in the neuroprotective effects exerted by BCH and that other EAAT carriers cannot compensate for EAAT3 loss in these settings. Of note, the knock‐down of EAAT3 did not affect the expression of either EAAT1 or EAAT2 in RA‐differentiated cells, confirming the specificity of the RNA silencing (Fig. S3A–D). Additionally, α‐syn plus rot treatment did not alter the expression of either EAAT1 or EAAT3 expression, while it increased EAAT2 expression in RA‐differentiated cells (Fig. S4C,D).

To verify whether the EAAT transporters located on the plasma membrane play any role in the neuroprotective effect of BCH in our PD model, we exposed cells to TFB‐TBOA (1 μm). In the presence of TFB‐TBOA, the ability of BCH to recover the drop of ATP synthesis after α‐syn plus rot treatment was not affected (Fig. 4D). Similarly, TFB‐TBOA treatment did not affect the improvement of cell viability induced by BCH (Fig. 4E,F). These results suggest that the plasma membrane EAAT transporters are not involved in the BCH‐dependent GDH activation.

### BCH‐dependent activation of GDH prevents mitochondrial ROS overproduction induced by α‐syn plus rot in RA‐differentiated SH‐SY5Y cells: Essential role of EAAT3 transporter

**Fig. 5:**
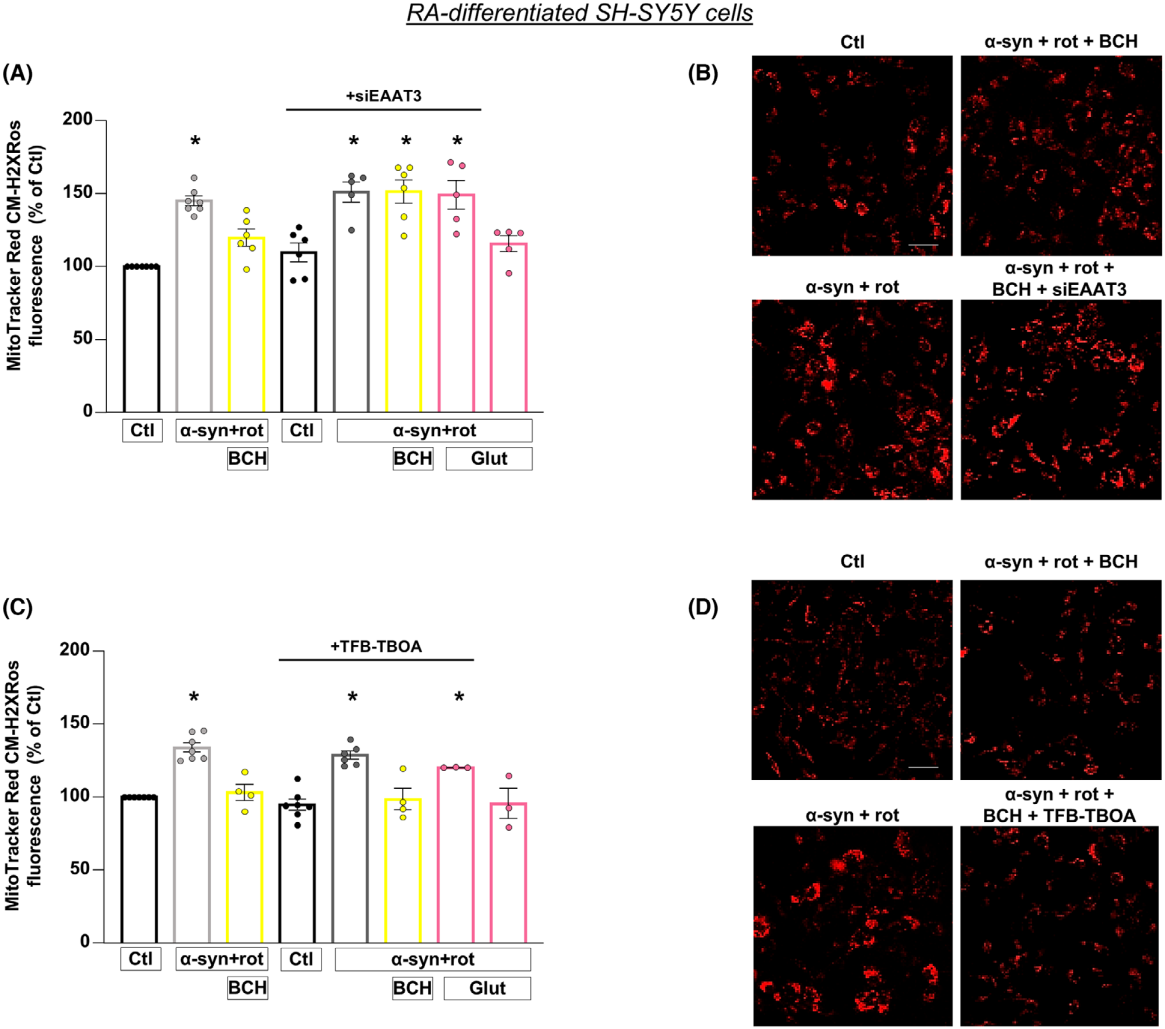
BCH‐dependent activation of GDH prevents mitochondrial ROS overproduction induced by α‐syn plus rot: essential role of EAAT3 transporter. Mitochondrial ROS generation was evaluated in α‐syn plus rot stressed cells that have been treated with/without BCH either after 48 h of EAAT3 silencing (panels A and B) or exposed to TFB‐TBOA (panels C and D). In both experimental series, Glut was used as internal control. Statistical differences among means were evaluated by one‐way ANOVA followed by Dunnett's *post hoc* test. Data are represented as means ± SEM. (A) *Significant vs Ctl (*P* < 0.0001), siEAAT3 (*P* < 0.001), α‐syn + rot + Glut (*P* < 0.01) and α‐syn + rot + BCH (*P* < 0.05). *n* = at least 5 independent experiments; (C) *Significant vs control groups, α‐syn + rot + BCH, α‐syn + rot + BCH + TFB and α‐syn + rot + Glut (*P* < 0.0001). *n* = at least 3 independent experiments; (B, D) Representative fluorescence images of mitochondrial ROS generation by staining with MitoTracker Red CM‐H2XRos deriving from at least 3 independent experiments. Scale bar = 50 μm.

As previously observed, α‐syn plus rot treatment dramatically affects oxidative stress [20, 40, 41, 42], therefore, the effect of BCH on mitochondrial ROS production was investigated in our experimental model. We found that BCH significantly reduced the increase in mitochondrial ROS production induced by α‐syn plus rot (Fig. 5). Of note, when cells were treated with siEAAT3, the protective effect of both BCH and Glut on mitochondrial ROS overproduction was fully prevented (Fig. 5A,B). On the contrary, TFB‐TBOA treatment was wholly ineffective in preventing the reduction in mitochondrial ROS production induced by BCH, whereas it completely counteracted the protective effect of Glut on mitochondrial ROS generation (Fig. 5C,D). These results highlight that the plasma membrane EAATs are not involved in the BCH effects thus supporting the hypothesis that the mitochondrial EAAT3 may exert a determinant role in the neuroprotection of BCH against α‐syn plus rot.

### BCH‐dependent activation of GDH prevents the drop of ΔΨ_m_ induced by α‐syn plus rot in RA‐differentiated SH‐SY5Y cells: Essential role of EAAT3 transporter

**Fig. 6:**
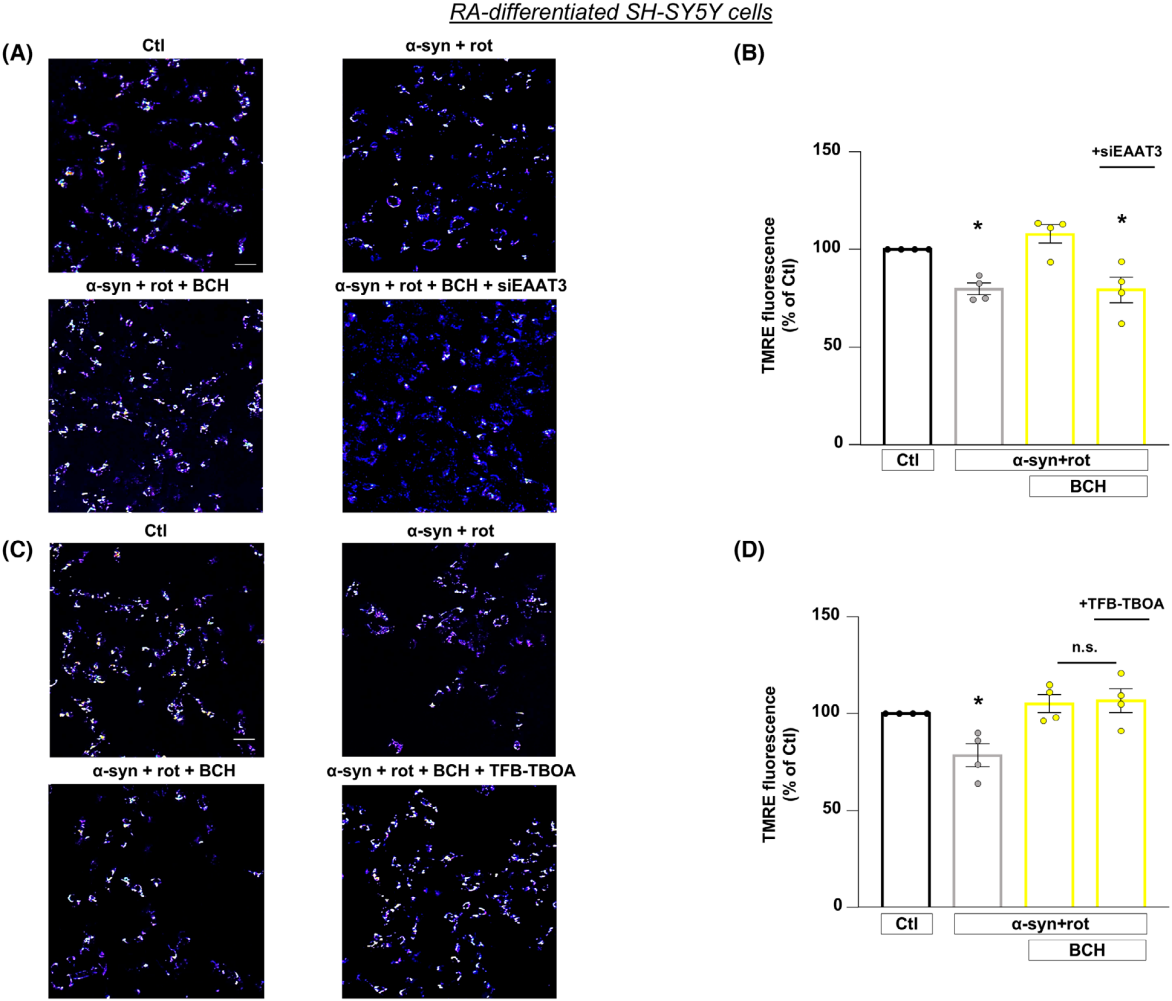
BCH‐dependent activation of GDH prevents the drop of mitochondrial membrane potential induced by α‐syn plus rot: essential role of EAAT3 transporter. (A, C) Representative pictures of mitochondrial membrane potential measurements under different experimental conditions. Scale bar 50 μm. Statistical differences among means were evaluated by one‐way ANOVA followed by Dunnett's *post hoc* test. (B) *Significant vs Ctl (*P* < 0.01) and α‐syn + rot + BCH (*P* < 0.001). Each column depicts the mean ± SEM of 50–100 cells recorded in 4 different sessions. (D) *Significant vs Ctl (*P* < 0.05), α‐syn + rot + BCH and α‐syn + rot + BCH + TFB‐TBOA (*P* < 0.01). Each column depicts the mean ± SEM of 50–100 cells recorded in 4 different sessions.

Since both α‐syn and rot are associated with various neurotoxic mechanisms including the disruption of ΔΨ_m_ [20, 43, 44], we explored the effect of BCH on ΔΨ_m_ after α‐syn plus rot treatment and evaluated the possible involvement of EAAT3. Data showed that α‐syn plus rot dramatically compromised mitochondrial function as reflected by the loss of ΔΨ_m_ (Fig. 6). Activation of GDH triggered by BCH prevented the drop of ΔΨ_m_ in RA‐differentiated SH‐SY5Y cells. To explore the possible involvement of EAAT3, silencing of EAAT3 was performed. The results obtained showed that the knock‐down of EAAT3 expression prevented the recovery of ΔΨ_m_ (Fig. 6A,B), while TFBOA treatment was ineffective in blocking BCH effect (Fig. 6C,D).

### BCH‐dependent activation of GDH in primary rat mesencephalic neurons requires EAAT3 transporters and confers neuroprotection against α‐syn plus rot toxicity

**Fig. 7:**
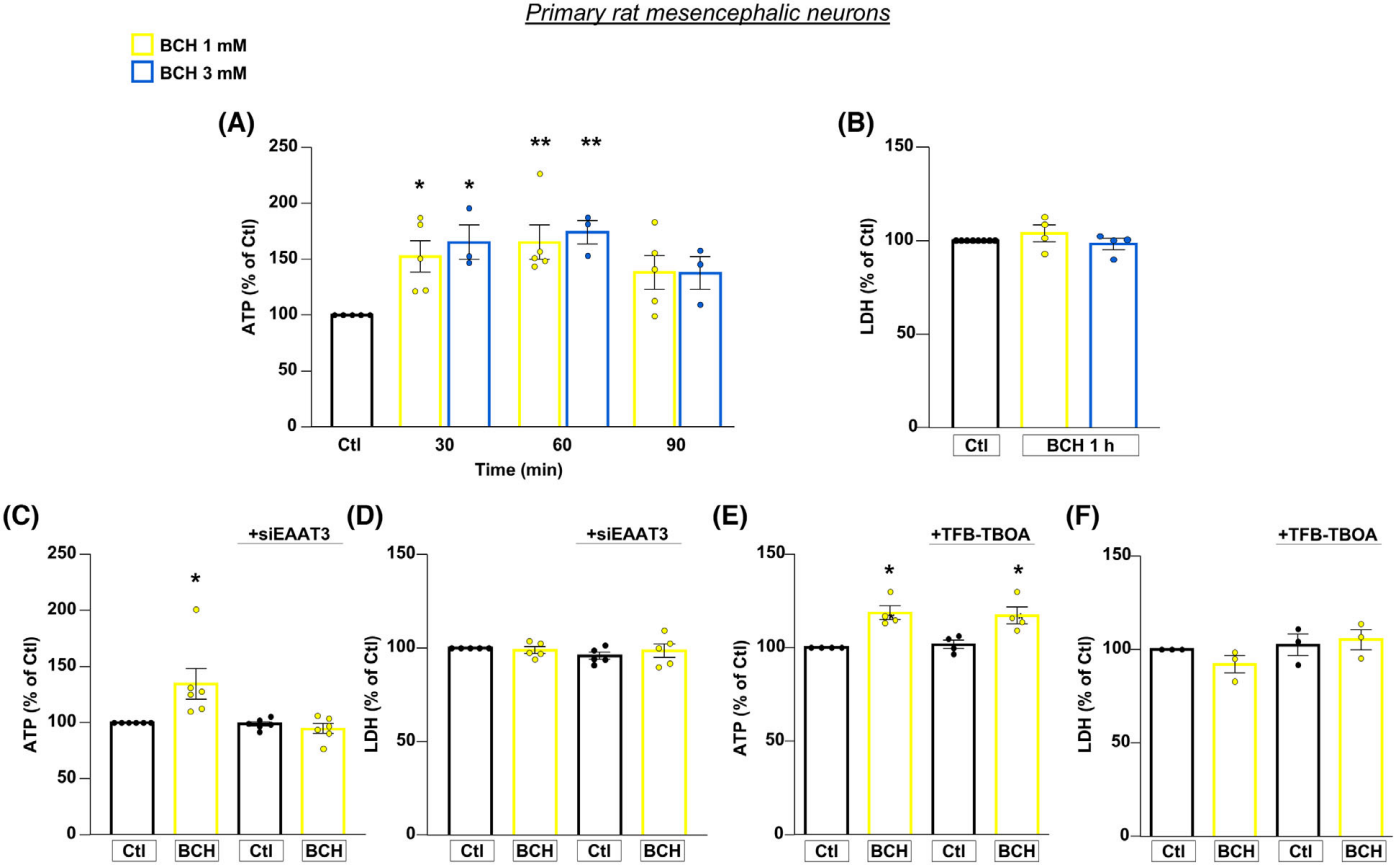
BCH‐dependent GDH activation promotes ATP synthesis in primary rat mesencephalic neurons: essential role of EAAT3 glutamate transporter. (A) ATP level was evaluated after 30, 60, 90 min exposure to BCH (1 or 3 mm). (B) Cell viability, measured by means of extracellular LDH activity, was evaluated after 1 h exposure to BCH (1 or 3 mm). (C, D) After knocking down of EAAT3 expression, intracellular ATP content (C) and cell viability (D) were measured after 1 h exposure to BCH (1 mm). (E, F) Intracellular ATP content (E) and cell viability (F) were measured after 30 min exposure to TFB‐TBOA (1 μm) followed by 1 h of BCH (1 mm). Statistical differences among means were evaluated by one‐way ANOVA followed by Dunnett's *post hoc* test. Data are represented as means ± SEM. (A) *Significant vs Ctl (*P* < 0.05); **Significant vs Ctl (*P* < 0.01). For 1 mm BCH *n* = 5 independent experiments; for 3 mm BCH 3 independent experiments; (B) *n* = 4 independent experiments; (C) *Significant vs all groups (*P* < 0.01). *n* = 6 independent experiments; (D) *n* = 5 independent experiments; (E) *Significant vs Ctl and TFB‐TBOA (*P* < 0.01). *n* = 4 independent experiments; (F) *n* = 3 independent experiments.

**Fig. 8:**
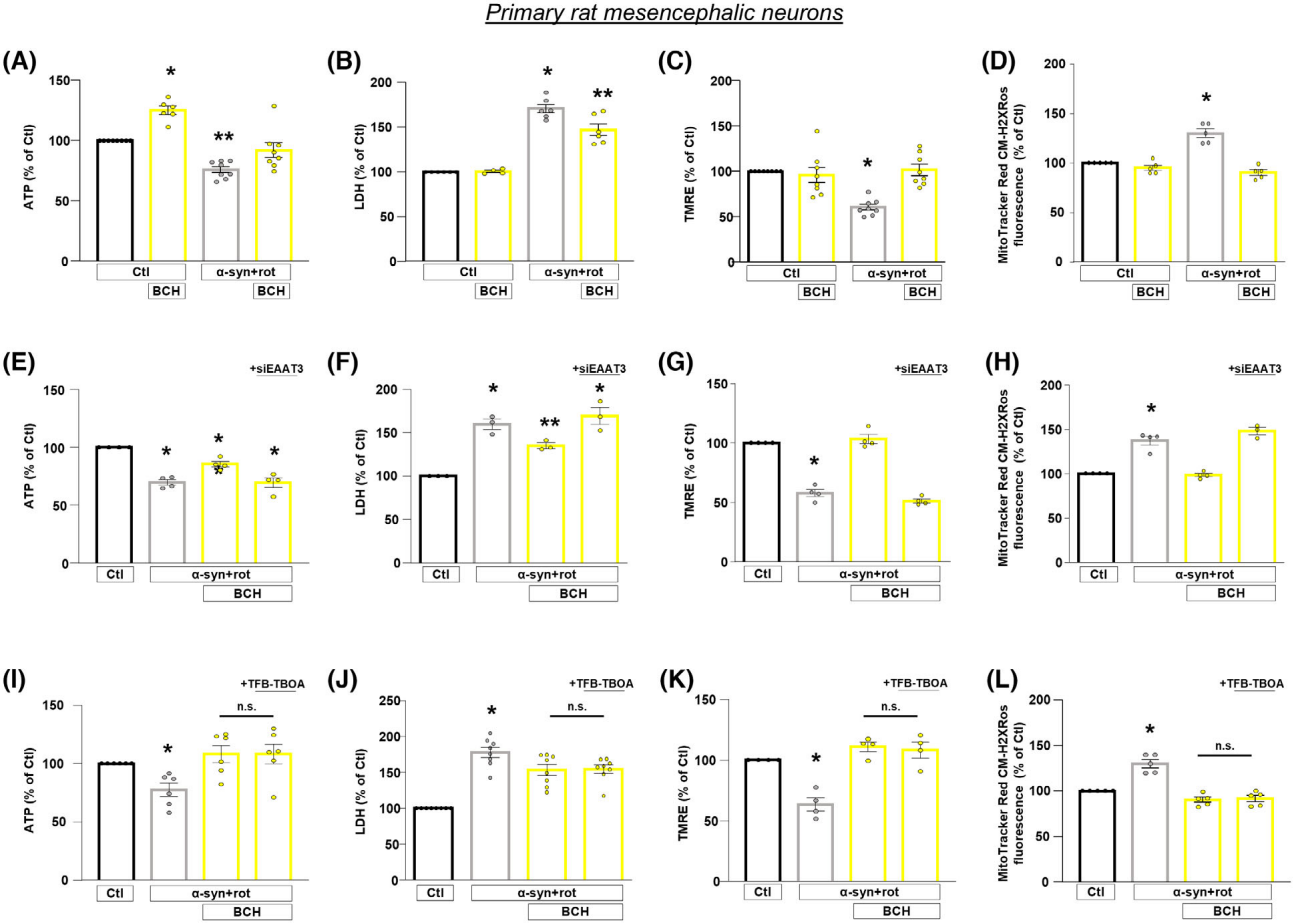
Involvement of EAAT3 glutamate transporters in the neuroprotective effects of BCH against α‐syn plus rot neurotoxicity in primary rat mesencephalic neurons. (A–L) Intracellular ATP levels (A, E, I), cell viability (B, F, J), mitochondrial membrane potential (C, G, K), and mitochondrial ROS production (D, H, L) were evaluated in cells exposed to α‐syn plus rot treated with/without BCH (1 mm) (A–D) either after 48 h of EAAT3 silencing (E–H) or exposed to TFB‐TBOA (1 μm) (I–L). Statistical differences among means were evaluated by one‐way ANOVA followed by Dunnett's *post hoc* test. Data are represented as means ± SEM. (A) *Significant vs all groups (*P* < 0.001 vs Ctl, *P* < 0.0001 vs α‐syn + rot and α‐syn + rot+BCH); **significant vs all groups (*P* < 0.001 vs Ctl, *P* < 0.0001 vs BCH, *P* < 0.05 vs α‐syn + rot+BCH). *n* = at least 6 independent experiments; (B) *Significant vs all groups (*P* < 0.0001 vs Ctl and BCH, *P* < 0.01 vs α‐syn + rot+BCH); **significant vs all groups (*P* < 0.0001 vs Ctl and BCH, *P* < 0.01 vs α‐syn + rot+BCH). *n* = at least 4 independent experiments; (C) *Significant vs all groups (*P* < 0.0001 vs Ctl and α‐syn + rot+BCH, *P* < 0.001 vs BCH). *n* = 8 independent experiments; (D) *Significant vs all groups (*P* < 0.0001). *n* = 5 independent experiments; (E) *Significant vs Ctl (*P* < 0.0001) and α‐syn + rot+BCH (*P* < 0.01); **significant vs all groups (*P* < 0.01). *n* = 4 independent experiments; F) *Significant vs Ctl (*P* < 0.001) and α‐syn + rot+BCH (*P* < 0.05); **significant vs all groups (*P* < 0.01 vs Ctl and α‐syn + rot+BCH + siEAAT3, *P* < 0.05 vs α‐syn + rot). *n* = 3 independent experiments; (G) *Significant vs Ctl and α‐syn + rot+BCH (*P* < 0.0001). *n* = 4 independent experiments; (H) *Significant vs Ctl and α‐syn + rot+BCH (*P* < 0.0001). *n* = at least 3 independent experiments; (I) *Significant vs all groups (*P* < 0.05 vs Ctl, *P* < 0.01 vs α‐syn + rot+BCH and α‐syn + rot+BCH + TFB‐TBOA). *n* = 6 independent experiments; (J) *Significant vs all groups (*P* < 0.0001 vs Ctl, *P* < 0.05 vs α‐syn + rot+BCH and α‐syn + rot+BCH + TFB‐TBOA). *n* = 8 independent experiments; (K) *Significant vs all groups (*P* < 0.001 vs Ctl, *P* < 0.0001 vs α‐syn + rot+BCH and α‐syn + rot+BCH + TFB‐TBOA). *n* = 4 independent experiments; (L) *Significant vs all groups (*P* < 0.0001). *n* = 5 independent experiments.

We further assessed the pathophysiological relevance of EAAT3/GDH functional axis in primary rat mesencephalic neurons exposed to α‐syn plus rot (Fig. 1). Notably, findings obtained in this model were fully consistent with those obtained in RA‐differentiated SH‐SY5Y cells, adding more weight to our results (Figs 7, 8; Figs S5, S6).

## Discussion

In this study, we explored a neuroprotective strategy focused on preserving neuronal bioenergetics, based on the hypothesis that defects in Glut metabolism are interconnected ends of a dysfunctional axis along which PD cell damage progresses. In particular, we found that the enhancement of GDH activity, by using BCH as a GDH activator, provides a neuroprotective effect against α‐syn plus rot‐induced neurotoxicity in both RA‐differentiated SH‐SY5Y cells and primary rat mesencephalic neurons. We therefore reasoned that the stimulation of mitochondrial metabolism, by promoting the utilization of Glut as a metabolic source, has a strong impact on preserving cellular functions, and our hypothesis is supported by the following findings: BCH‐dependent GDH activation significantly improved mitochondrial ATP synthesis, lessened to control levels the cellular redox burden and prevented the drop of mitochondrial membrane potential, thereby limiting the overall cell damage.

An efficient energy supply is critical for the optimal functioning of the brain: any deviation from a regulated pattern of ATP production and use may incite cell dysfunction and loss in neurodegenerative disorders as PD, especially when this occurs within a compromised metabolic landscape [45]. Although there is a solid and general consensus that mitochondrial bioenergetic deficiency has a prominent role in PD pathogenesis and represents an early biomarker for neurodegenerative conditions [46], we are still on the road to figuring out how to best preserve bioenergetic to halt the neurodegenerative spiral.

Previous studies reported that mitochondrial ATP synthesis fueled by extracellularly applied Glut translates into substantial benefits for Ca^2+^ homeostasis, redox control and cell survival in different models of brain disorders, including *in vitro* models of brain ischemia, PD and Alzheimer disease (AD) like models [20, 32, 47]. Here, we documented that a complementary approach promoting the metabolic use of endogenous Glut is beneficial against PD‐related injury and may have therapeutic potential because it can simultaneously address two key issues at once: Glut‐mediated excitotoxicity and the upstream energetic failure. Indeed, we showed that BCH treatment increased the mitochondrial GDH activity thus promoting the Glut influx into the TCA cycle and enhancing the intracellular ATP content. This bioenergetics stimulation positively affected cellular viability protecting cells from the energy failure induced by α‐syn plus rot treatment. Moreover, the improvement of energy metabolism reduced oxidative stress and recovered the drop of mitochondrial membrane potential. In line with these results, in both *in vivo* and *in vitro* models of cerebral ischemia it was observed how the modulation of mitochondrial GDH activity rescued neuronal death by restoring the intracellular ATP level through the conversion of Glut to α‐ketoglutarate [29]. Additionally, it has been reported that the mitochondrial GDH1 isoform is tightly involved in maintaining redox homeostasis through the regulation of α‐ketoglutarate levels and those of fumarate, its downstream metabolite [48]. Specifically, Jin and colleagues demonstrated that fumarate acts as a signaling molecule which controls glutathione peroxidase 1 activity, enhancing the ROS‐scavenging ability of the cells [48]. A similar mechanism may play a role in the BCH‐dependent reduction of ROS production in our settings.

The association between GDH deficiency and neurodegeneration has been documented in the literature [49, 50, 51], which is consistent with our findings. However, a previous report provided evidence that an overactive variant of the GDH2 isoform, which is predominantly expressed in astrocytes and to a lesser extent in neurons [52, 53], is linked to early clinical signs of PD [54]. In this condition, astrocytes would divert Glut to glial oxidative use at expense of its extracellular release and share with neurons via glutamine‐glutamate cycle. Either when GDH activity is globally reduced or more pronounced in astrocytes, the functional and metabolic astrocyte‐neuronal coupling can be significantly compromised. In both scenarios, without a delivery of Glut from astrocytes that is optimized for the bioenergetic needs of neurons, the GDH‐deficient neurons would be the more vulnerable cells because neurons are incapable of an efficient *de novo* synthesis of Glut [55].

Considering the distinct roles of GDH1 and GDH2 isoforms in pathophysiology and the challenge of selectively targeting each isoform pharmacologically [56, 57], stimulating GDH activity is a complex process that highlights the need for precise regulation of GDH activity.

EAATs transporters are the major transport mechanisms for extracellular Glut removal in the central nervous system [21, 58]. These transporters regulate the utilization of Glut to efficiently perform various cellular functions, including neurotransmission, prevention of excitotoxicity, and regulation of the cellular redox state and energy metabolism [20, 21, 47]. Therefore, we investigated the possible involvement of EAATs in the BCH metabolic response, and we found that BCH‐dependent GDH activation is strongly associated with EAATs activity. Considering the key role of EAATs in Glut transport as well as the fact that they are expressed at both plasma membrane and mitochondrial levels [21, 58] of particular interest is our observation that silencing of EAAT3, but not the inhibition of plasma membrane EAATs with TFB‐TBOA, fully prevented the BCH‐induced neuroprotection in our PD model. These results suggest the existence of a functional axis connecting GDH activity to a specific intracellular pool of EAATs transporters, namely EAAT3. In particular, we hypothesized that among the intracellular pool of EAATs transporters, the mitochondrial EAAT3 may be critically involved in the pro‐survival effect of BCH. Recent studies conducted by our research group reported that the knock‐down of EAAT3 abolished the neuroprotection induced by Glut in different pathological settings, such as PD‐like model, AD‐like models and a model of neuronal hypoxia‐reoxygenation injury [20, 32, 47]. Specifically, the neuroprotective effects of Glut have been closely linked to the expression of EAAT3. Consistent with these findings, other studies reported that in animal models of PD, strategies aimed at enhancing both the expression and activity of EAATs improved cognitive functions and neuronal viability in the substantia nigra and striatum [24].

Although the activity of EAATs transporters is crucial in regulating the extracellular concentration of Glut by facilitating its transport across cellular membranes [21], it may not be sufficient to ensure the effective entry of Glut into cells and, subsequently, into the mitochondria. Therefore, stimulating the mitochondrial GDH activity could simultaneously enhance the uptake of Glut into the mitochondria (and, thereby facilitating its bioenergetic use) and reduce the extracellular level of Glut responsible for excitotoxic damage.

To conclude, we demonstrated that BCH stimulates GDH activity in dopaminergic neuronal cells, boosting Glut metabolism and providing neuroprotection against α‐syn plus rot‐induced toxicity. We also observed that silencing of EAAT3 significantly prevented the metabolic response of BCH, indicating that EAAT3 plays a crucial and non‐redundant role in GDH‐mediated ATP synthesis in a PD model. These findings suggest that therapeutic strategies targeting the pre‐symptomatic stages of PD could offer a substantial advantage over conventional dopaminergic replacement therapies, which primarily address late‐stage symptoms, or current pharmacotherapies that focus on downstream excitotoxic effects while overlooking underlying energy deficiencies. However, given that GDH also influences insulin regulation in pancreatic β cells, its persistent overactivation could lead to hyperinsulinemia and hypoglycemia [59]. Therefore, precise regulation of GDH activity is crucial.

This proof of concept highlights the relevant role of EAAT3/GDH functional axis in promoting neuroprotection in a PD model. Additional experiments are needed to validate these findings in more complex models incorporating astrocytic components. Such studies are essential for gaining a comprehensive understanding of the role of astrocytes and their metabolic interactions with neurons in PD pathogenesis.

### Limitations of the study

Although our results indicate that the stimulation of GDH activity can improve mitochondrial function, thereby limiting cellular damage following treatment with α‐syn plus rot, some issues are still open and require further consideration. First, whether GDH activity, sustained by GDH1 and/or GDH2, has protective or detrimental impact on brain cells has been not unequivocally established for all neurodegenerative disorders exanimated. This may simply indicate that the role of GDH function in the metabolic collaboration between neurons and astrocytes depends on the type of neurodegenerative pathology considered [60, 61, 62] and/or the severity of its clinical expression. Second, we used BCH as pharmacological tool to stimulate GDH activity [29, 61, 63]. To minimize off target effects of BCH, we used a rather low concentration of BCH (1 mm) for 1 h as done in previous study [29, 64, 65, 66]. Nevertheless, more specific activators of GDH1 or of GDH2, which are currently not commercially available, will be required to shed more light on the potential of stimulating GDH activity against PD neurodegenerative damage. Third, we used a “chemically” induced PD model (i.e., relying on α‐syn plus rot) on otherwise healthy neurons. To further establish our findings in a model system that is less dependent on physiological manipulation, future work will be required using iPSC/iNPC models derived from people living with PD.

## Materials and methods

### Cell culture and treatment

The human neuroblastoma cell line SH‐SY5Y (RRID: CVCL_0019, as listed in the ExPASy Cellosaurus database) (American Type Culture Collection, MD, USA) (CRL‐2266) were cultured as a monolayer and grown in polystyrene dishes (100 mm diameter) in Dulbecco's modified Eagle's medium (DMEM; Corning, New York, NY, USA) containing 100 U·mL^−1^ penicillin, and 100 μg·mL^−1^ streptomycin and supplemented with 10% fetal bovine serum (FBS) (Corning). The methods for cell culture and treatment have been previously described [20, 67, 68] and are presented here with minor modifications for clarity. Cells were maintained in an atmosphere of 5% CO_2_, at 37 °C, in a humidified incubator. Cell culture medium was replaced every 2 days. Neuronal differentiation was induced by treating cells with 10 μm all‐trans retinoic acid (RA) for 7 days [67]. Cells were challenged for 24 h with α‐syn (10 nm) (the generation of α‐syn oligomers was previously described in [20] plus Rot (300 nm) (Sigma‐Aldrich, St. Louis, MO, USA), BCH (1 mm) (Sigma‐Aldrich) was added during the last hour of the treatment). At the end of the 24 h of the experimental protocol, cell viability (assessed by extracellular lactate dehydrogenase (LDH) activity and MTT assay), ATP production, mitochondrial ROS generation and mitochondrial membrane potential analysis were performed. Cells were plated on 6‐well plates with round glass coverslips (140 000 cells·well^−1^) for the evaluation of mitochondrial ROS production. Cell viability and mitochondrial activity were performed on cells plated on 12‐well plates (70 000 cells·well^−1^), while for the detection of intracellular ATP content cells were plated on 96‐well plates (15 000 cells·well^−1^). All experiments were conducted using SH‐SY5Y cells confirmed to be free of Mycoplasma contamination. Mycoplasma testing was performed regularly using the Platinum™ SuperFi II PCR Master Mix (Thermo Fisher Scientific, Waltham, MA, USA), following the manufacturer's instructions, and no contamination was detected.

Primary rat mesencephalic neurons were extracted from the midbrains of Wistar rat pups (P2–P4) (Cat. 003WISTAR, Charles River, Lecco, Italy) [69]. The animal use and procedures fully adhered to the protocols approved by the Ethics Committee for Animal Experiments at the University “Politecnica delle Marche” and complied with the Italian Ministry of Health guidelines (D.L. 26/2014). The Ethics Committee for Animal Experiments at the University “Politecnica delle Marche” approved this study on 22‐01‐2021, with the reference number 40A31.N.IDH. Rats were housed in specific pathogen‐free facilities, where rodent care was conducted under sterile conditions with the use of sterilized supplies. The housing environment featured a 12:12 light–dark cycle, maintained at temperatures of 20–24°C and relative humidity levels of 30–70%. The animals had continuous access to autoclaved rodent chow and reverse osmosis‐filtered water.

Briefly, the midbrains were quickly placed in ice‐cold PBS following isolation and then subjected to trypsinization (0.05% trypsin/EDTA) for 15 min at 37 °C. After further homogenization, the neurons were collected and plated on poly‐D‐lysine‐coated plates on 12 well plates (6 × 10^5^ cells·well^−1^) for cell viability, ATP production were performed. For mitochondrial ROS generation and mitochondrial membrane potential analysis, cells were cultured on poly‐d‐lysine‐coated glass coverslips on 6 well plates (1.3 × 10^6^ cells·well^−1^). Cultures were maintained at 37 °C in a humidified atmosphere of 5% CO_2_ in Neurobasal medium (Gibco‐Invitrogen, Paisley, UK) supplemented with B27 (Gibco‐Invitrogen) and 2 mm glutamine in the presence of penicillin/streptomycin. Medium was changed twice a week, and experiments were performed between 10 and 16 DIV (days *in vitro*). Cells were challenged for 24 h with α‐syn (10 nm) plus Rot (300 nm), BCH (1 mm) was added during the last hour of the treatment.

### Silencing of EAAT3

Silencing of EAAT3 was performed as previously described [20, 32]. Briefly, RA‐differentiated cells and rat primary mesencephalic neurons were plated on 12‐well plates and were incubated for 48 h with 80 nm of EAAT3 siRNA oligonucleotide (each well). RNA interference was performed with HiPerfect Transfections Kit (Qiagen) according to the manufacturer's instruction by using FlexiTube siRNA for human EAAT3 (Qiagen Hs_SLC1A1_3; target sequence 5′‐GAGGACTGTTCTAACTAGTAA‐3′) and FlexiTube siRNA for rodent EAAT3 (Qiagen Rn_SLC11A1_3; target sequence 5′‐CACTACTTCTATTAAACCGAA‐3′). After 48 h of siRNA transfection, cells were subjected to experimental treatment. Then, cells were tested for viability, ATP content, mitochondrial ROS production and mitochondrial membrane potential. The efficacy of EAAT3 RNAi approach was verified by measuring the protein expression level. For RA‐differentiated SH‐SY5Y cells, EAAT3 level was reduced by ~50% (as described in [32]); for primary rat mesencephalic neurons, EAAT3 level was reduced by ~40% (Fig. S5).

### Cell viability assay

Cell viability was detected by measuring the lactate dehydrogenase (LDH) activity released into the culture supernatant [70]. After the experimental protocols, 50 μL of supernatant of RA‐differentiated SH‐SY5Y was incubated with 50 μL of reaction mixture (Roche Diagnostics, Monza, Italy) for 30 min at room temperature in the dark. The released LDH from damage cells was measured by reading the absorbance at 490 nm in a Victor Multilabel Counter plate reader (Perkin Elmer, Waltham, MA, USA). The results were expressed as percentages of the control value.

### Mitochondrial activity

Mitochondrial activity was evaluated by using 3‐(4,5‐dimethylthiazol‐2‐yl)‐2,5‐diphenyltetrazolium bromide (MTT) assay. This colorimetric assay is based on the ability of viable cells to reduce the yellow tetrazolium salt MTT to purple insoluble crystals of formazan. At the end of the experimental procedure, cells were incubated with 0.5 mL of MTT solution (0.5 mg·mL^−1^ in PBS) at 37 °C and in a 5% CO_2_ atmosphere in a humidified incubator, in the dark. After 1 h, the produced formazan crystals were collected, centrifugated and dissolved in 0.5 mL of DMSO [47]. Absorbance was read at a wavelength of 540 nm using a Victor Multilabel Counter plate reader (Perkin Elmer, Waltham, MA, USA). The results were expressed as percentages of the control value.

### ATP assay

The intracellular content of ATP was measured by using a commercially available luciferase‐luciferin system (ATPlite, Perkin Elmer). Briefly, RA‐differentiated SH‐SY5Y were plated in 96‐well “ViewPlate” (Perkin Elmer) and subjected to the experimental protocol in DMEM medium and then analyzed for the ATP content as reported by the manufacturer's protocol. Primary rat mesencephalic neurons were cultured in 12‐well plates, treated with Neurobasal medium according to the designated experimental groups, and subsequently lysed. The cell lysates were then transferred to a 96‐well ViewPlate (Perkin Elmer) for an ATP assay. Intracellular ATP levels were measured using a luminescence counter (Victor Multilabel Counter, Perkin Elmer), normalized to the corresponding protein content, and reported as percentages relative to the control value [20].

### Evaluation of mitochondrial ROS production

Mitochondrial ROS production was analyzed by using mitochondrial‐targeted dye MitoTracker CM‐H2XRos (Invitrogen Life Technologies, Carlsbad, CA, USA) as previously described [71]. At the end of the experimental treatment, cells were loaded with the dye (300 nm) for 30 min, at 37 °C, in the dark. Confocal images were collected using a 510 LSM microscope (Carl Zeiss) with a META detection system. Fluorescence excitation and emission of CM‐H2XRos was 560 ± 10 nm and 620 ± 20 nm, respectively. Analysis of fluorescence intensity was performed offline after image acquisition. A random selection of 3 to 5 images was taken from each coverslip. For each repetition, 50 to 100 cells were analyzed in at least 3 images, and the average was calculated for each repetition. The fluorescence values are expressed as percentages relative to the control values.

### Measurement of ΔΨ_m_

ΔΨ_m_ was assessed by measuring the fluorescence of tetramethylrhodamine ethylester (TMRE, Abcam, Cambridge, UK) in a non‐quenching mode [68, 72]. RA‐differentiated SH‐SY5Y cells and primary rat mesencephalic neurons were plated on a poly‐d‐lysine‐coated glass coverslips and treated by following the experimental protocols. Cells were incubated with 10 nm TMRE in the culture medium at 37 °C for 30 min, followed by two PBS washes. Subsequently, they were permeabilized for 1 min using an intracellular buffer with 5 μm digitonin and 10 nm TMRE. The buffer composition (in mm) of RA‐differentiated SH‐SY5Y cells was as follows: 5 NaCl, 1 MgCl_2_, 25 Sucrose, 10 KH_2_PO_4_, 10 Tris/HCl, 0.0001 CaCl_2_, 70 Mannitol, 5.5 Glucose, 2 Pyruvate, 2 Malate, 3 Succinate, 0.1 ADP. The buffer composition (in mm) of primary rat mesencephalic neurons was as follows: 135 KCl, 10 NaCl, 20 HEPES, 5 pyruvate, 2 glutamate, 2 malate, 0.5 KH_2_PO_4_, 1 MgCl_2_, 5 EGTA, and 1.86 CaCl_2_. Digitonin at low concentrations selectively permeabilize the plasma membrane without affecting the integrity of organelles such as mitochondria. After permeabilization, cells were continuously perfused with an intracellular solution containing 10 nm TMRE and 5 μm digitonin. In this setup, a reduction in TMRE fluorescence signifies mitochondrial membrane depolarization. Images were captured every 5 s using a 510 LSM microscope (Carl Zeiss), with TMRE excitation at 543 nm and emission detected between 580 and 700 nm. Baseline ΔΨm levels were recorded for approximately 300 s. After 180 s, 20 μm carbonyl cyanide p‐trifluoromethoxyphenylhydrazone (FCCP) was added as an internal control. The fluorescence intensity data were analyzed offline following image acquisition. For each experimental condition, 50 to 100 cells were analyzed in at least 3 independent experiments. The fluorescence values are reported as percentages relative to the control values.

### Statistical analysis

Data were reported as the mean ± standard error of the mean (SEM). GraphPad Prism® 5 software (San Diego, CA, USA) was used for the statistical analyses of the results. One‐way ANOVA analysis followed by Dunnett's *post hoc* test or Student's *t*‐test were applied to determine the differences between the experimental groups. Differences were considered statistically significant when *P* < 0.05.

## Conflict of interest

The authors declare no conflict of interest.

## Author contributions

VL and SP conceived and planned the experiments. AP, TS, MT, VT, SA, SM, SP contributed to data acquisition and analysis. AP, VL and SP wrote the manuscript. AP, TS, MT, VT, SM, VL and SP contributed to the interpretation of the results. VL acquired funding. All authors reviewed and edited the manuscript and approved the final version of the manuscript submitted for publication.

## Peer review

The peer review history for this article is available at https://www.webofscience.com/api/gateway/wos/peer‐review/10.1111/febs.70053.

## Supporting information


**Fig. S1.** Effect of both EAAT3 inhibition and knock‐down on cell viability in RA‐differentiated SH‐SY5Y cells.
**Fig. S2.** Effect of 10 mm 2‐DG on BCH‐induced the increase in ATP synthesis in RA‐differentiated SH‐SY5Y cells.
**Fig. S3.** Evaluation of EAAT3 silencing on both EAAT1 and EAAT2 expression in RA‐differentiated SH‐SY5Y cells.
**Fig. S4.** Effect of α‐synuclein plus rotenone treatment on EAATs expression in RA‐differentiated SH‐SY5Y cells.
**Fig. S5.** Effect of EAAT3 silencing on EAATs expression in primary rat mesencephalic neurons.
**Fig. S6.** Effect of α‐synuclein plus rotenone treatment on EAATs expression in primary rat mesencephalic neurons.

## Data Availability

The data that support the findings of this study are available from the corresponding author upon reasonable request.
