# Supplementary material for: A non‐redundant role of EAAT3 for ATP synthesis mediated by GDH in dopaminergic neuronal cells: a new avenue for glutamate metabolism and protection in Parkinson's disease
Source: FEBS J. 2025 Mar 5;292(12):3224–41. doi: 10.1111/febs.70053 (PMC12176255; doi:10.1111/febs.70053)
Supplement: Supplementary file 1 — Fig. S1. Effect of both EAAT3 inhibition and knock‐down on cell viability in RA‐differentiated SH‐SY5Y cells. Fig. S2. Effect of 10 mm 2‐DG on BCH‐induced the increase in ATP synthesis in RA‐differentiated SH‐SY5Y cells. Fig. S3. Evaluation of EAAT3 silencing on both EAAT1 and EAAT2 expression in RA‐differentiated SH‐SY5Y cells. Fig. S4. Effect of α‐synuclein plus rotenone treatment on EAATs expression in RA‐differentiated SH‐SY5Y cells. Fig. S5. Effect of EAAT3 silencing on EAATs expression in primary rat mesencephalic neurons. Fig. S6. Effect of α‐synuclein plus rotenone treatment on EAATs expression in primary rat mesencephalic neurons. [file FEBS-292-3224-s001.pdf]

Materials and methods

Western blot analysis

Western blot experiments were conducted on total lysates of RA-differentiated SH-SY5Y cells and primary rat mesencephalic neurons [72]. Cell lysates were prepared in B-buffer 1X containing (in mM): 150 NaCl, 10 Tris- HCl (pH 7.4), 1 EDTA (pH 8.0), 1% SDS. Protein content was assessed by the Bradford method (Bio-Rad, Milan, Italy). Samples containing equal amounts of protein (40 µg) were prepared in 6× Laemmli sample buffer with 2–mercaptoethanol and boiled for 10 min. Proteins were separated through electrophoresis onto an 8% SDS polyacrylamide gel and electro-transferred to a nitrocellulose membrane (Bio-Rad). To reduce nonspecific interactions, the membranes were blocked with non-fat dry milk (5% in PBS buffer), for 1 h at room temperature. After the blocking phase, the membranes were incubated with primary antibody overnight at 4 °C. To detect the immunoreactions, membranes were incubated with the appropriate secondary antibody conjugated to horseradish peroxidase (Goat anti-rabbit IgG-HRP Cat. sc-2004, Santa Cruz; Goat anti-Mouse IgG (H + L) HRP Cat. 62-6520, Thermo Scientific) for 1 h at room temperature. Then, blots were developed with an enhanced chemiluminescence detection kit (Super Signal West Femto kit, Thermo Scientific), and images were acquired with a Uvitec Cambridge Chemiluminescence Imaging System (Cambridge, UK).

Primary antibodies.

Primary antibodies used are listed below: anti-GLAST/EAAT1 (Cat. AB-82224, diluition 1:1000, Immulogical Sciences), anti-GLT-1/EAAT2 (Cat. AB-82459, dilution 1:1000, Immulogical Sciences), anti-EAAC1/EAAT3 (Cat. AB-82034, dilution 1:1000, Immulogical Sciences), anti-β-actin (Cat. sc-47778, dilution 1:1000, Santa Cruz). Band densities were analyzed with Uvitech Nine Alliance analysis software (Cambridge, UK) and normalized to the appropriate housekeeping protein expression.

Supplementary figures and figure legends

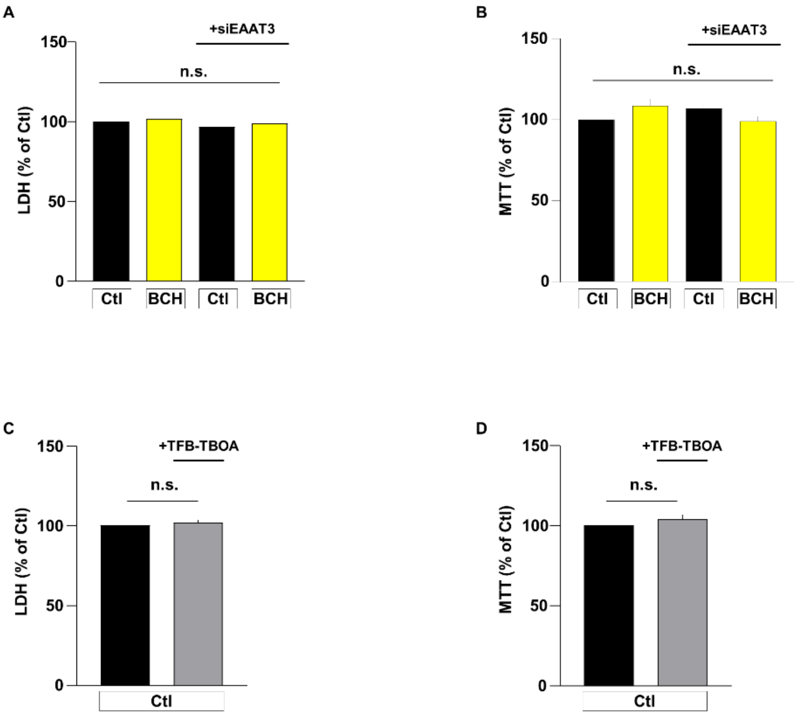

Figure S1. Effect of both EAAT3 inhibition and knockdown on cell viability in RA-differentiated SH-SY5Y cells. A, B) After silencing of EAAT3 expression, cell viability by measuring LDH activity (A) and MTT assay (B) were evaluated after 24 h exposure to α-syn plus rot and 23 h exposure to α-syn plus rot followed by 1 h of BCH (1 mM) in RA-differentiated SH-SY5Y cells. C, D) After 1.5 h of TFB-TBOA exposure, cell viability by measuring LDH activity (C) and MTT assay (D) were evaluated under physiological condition. In each experiment both LDH and MTT values were reported as percentages of the control value. Each column represents the mean ± S.E.M. of at least 3 independent experiments. Differences among means were assessed by one-way ANOVA followed by Dunnett’s post hoc test or Student’s t-test.

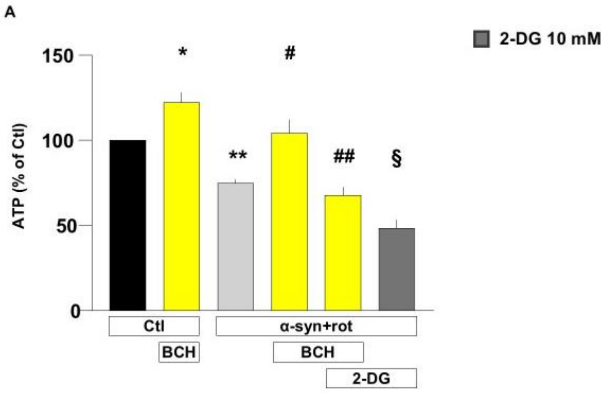

Figure S2. Effect of 10 mM 2-DG on BCH-induced the increase in ATP synthesis in RA-differentiated SH-SY5Y cells. *Significant vs Ctl ($p < 0.05$), vs α-syn + rot, α-syn + rot + BCH + 2-DG and α-syn + rot + 2-DG ($p < 0.0001$); ** significant vs Ctl and α-syn + rot + 2-DG ($p < 0.01$), vs BCH ($p < 0.0001$) and vs α-syn + rot + BCH ($p < 0.001$); # significant vs α-syn + rot ($p < 0.001$), vs α-syn + rot + BCH + 2-DG and α-syn + rot + 2-DG ($p < 0.0001$); ## significant vs Ctl ($p < 0.001$), vs BCH and α-syn + rot + BCH ($p < 0.0001$) and vs α-syn + rot + 2-DG ($p < 0.05$); § significant vs all groups ($p < 0.0001$ vs Ctl, BCH and α-syn + rot + BCH, $p < 0.01$ vs α-syn + rot, $p < 0.05$ vs α-syn + rot + BCH + 2-DG). n = at least 5 independent experiments.

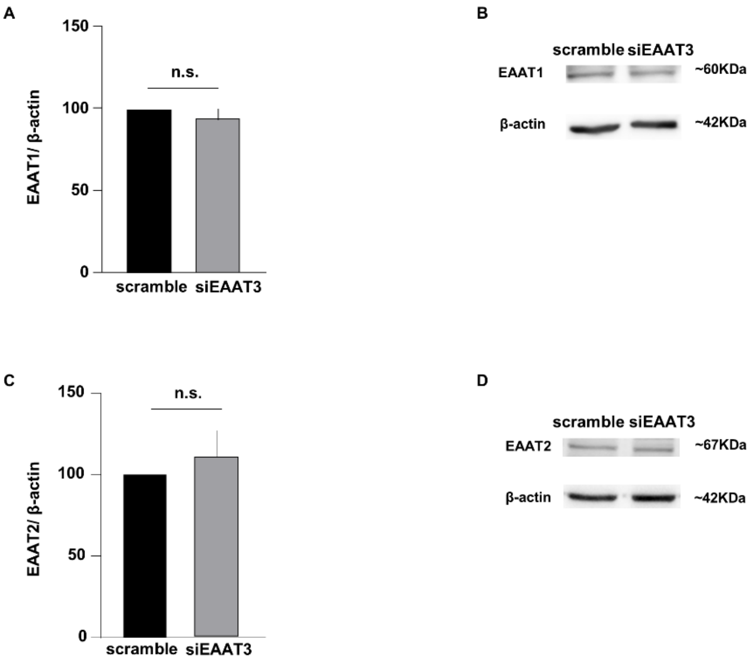

Figure S3. Evaluation of EAAT3 silencing on both EAAT1 and EAAT2 expression in RA-differentiated SH-SY5Y cells. A, C) Quantitative analysis of EAAT1 (A) and EAAT2 (C) after 48 h of EAAT3 silencing in RA-differentiated SH-SY5Y cells. B, D) Representative western blot images of EAAT1 (B) and EAAT2 (D) expression. Normalized optical density values are expressed as percentage of the respective control. Each column represents the mean ± S.E.M. of at least 3 independent experiments. Differences among means were assessed by Student's t-test.

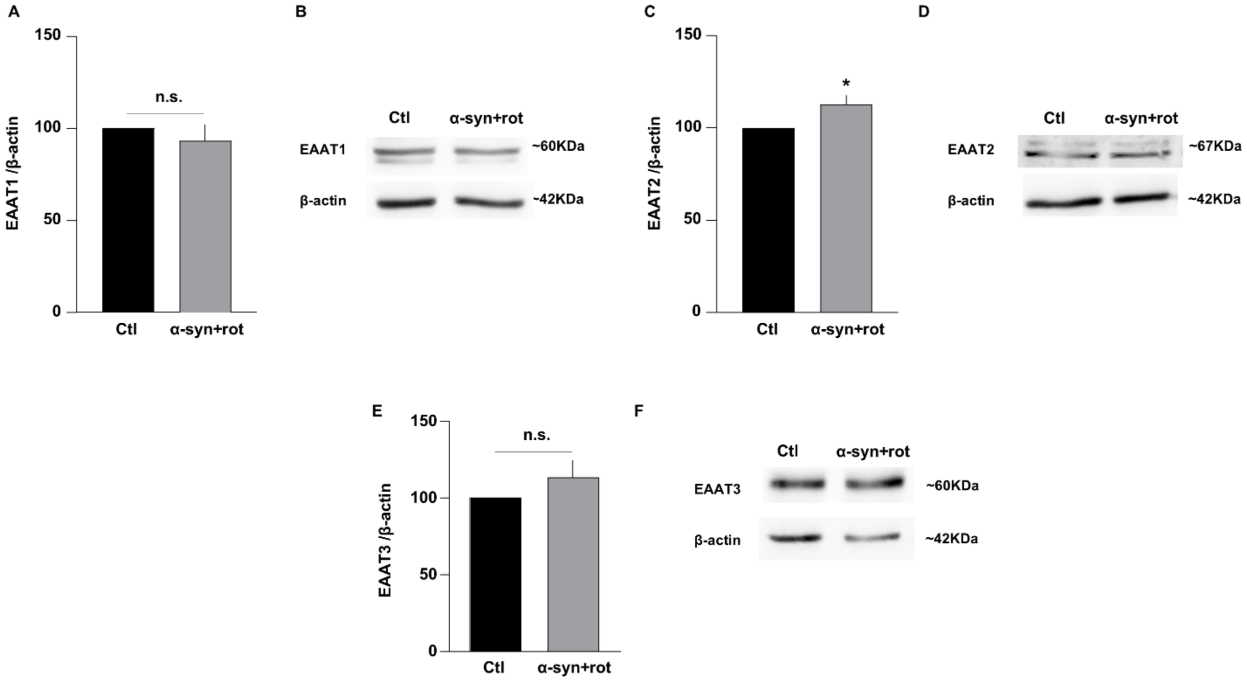

Figure S4. Effect of α-synuclein plus rotenone treatment on EAATs expression in RA-differentiated SH-SY5Y cells. A, C, E) Quantitative analysis of EAATs expression after α-syn + rot treatment in RA-differentiated SH-SY5Y cells. B, D, F) Representative western blot images of EAAT1 (B), EAAT2 (D) and EAAT3 (F) expression. Normalized optical density values are expressed as percentage of the respective control. Each column represents the mean ± S.E.M. of at least 3 independent experiments. Differences among means were assessed by Student's t-test. C) *Significant vs Ctl ($p < 0.05$).

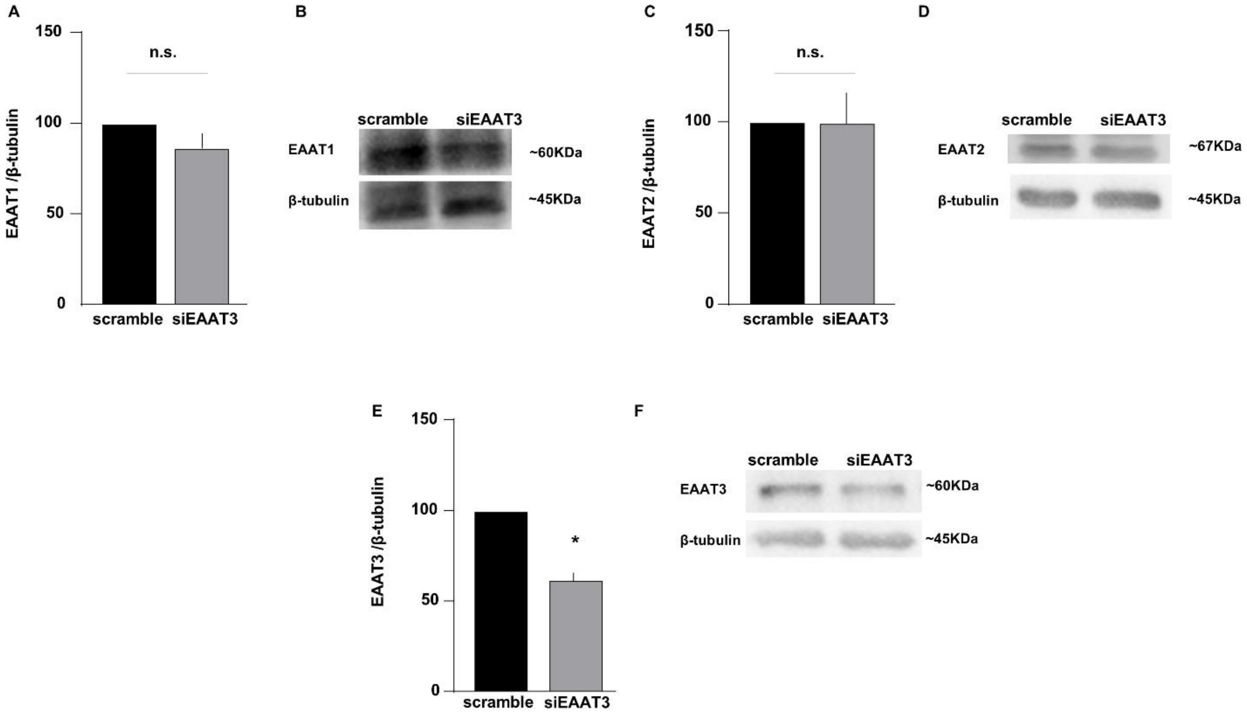

Figure S5. Effect of EAAT3 silencing on EAATs expression in primary rat mesencephalic neurons. A, C, E) Quantitative analysis of EAATs expression after 48 h of EAAT3 silencing in primary mesencephalic neurons. B, D, F) Representative western blot images of EAAT1 (GLAST is the rodent homologue) (B), EAAT2 (GLT-1 is the rodent homologue) (D) and EAAT3 (EAAC1 is the rodent homologue) (F) expression. Normalized optical density values are expressed as percentage of the respective control. Each column represents the mean ± S.E.M. of at least 3 independent experiments. Differences among means were assessed by Student's t-test. E) *Significant vs Ctl ($p < 0.0001$).

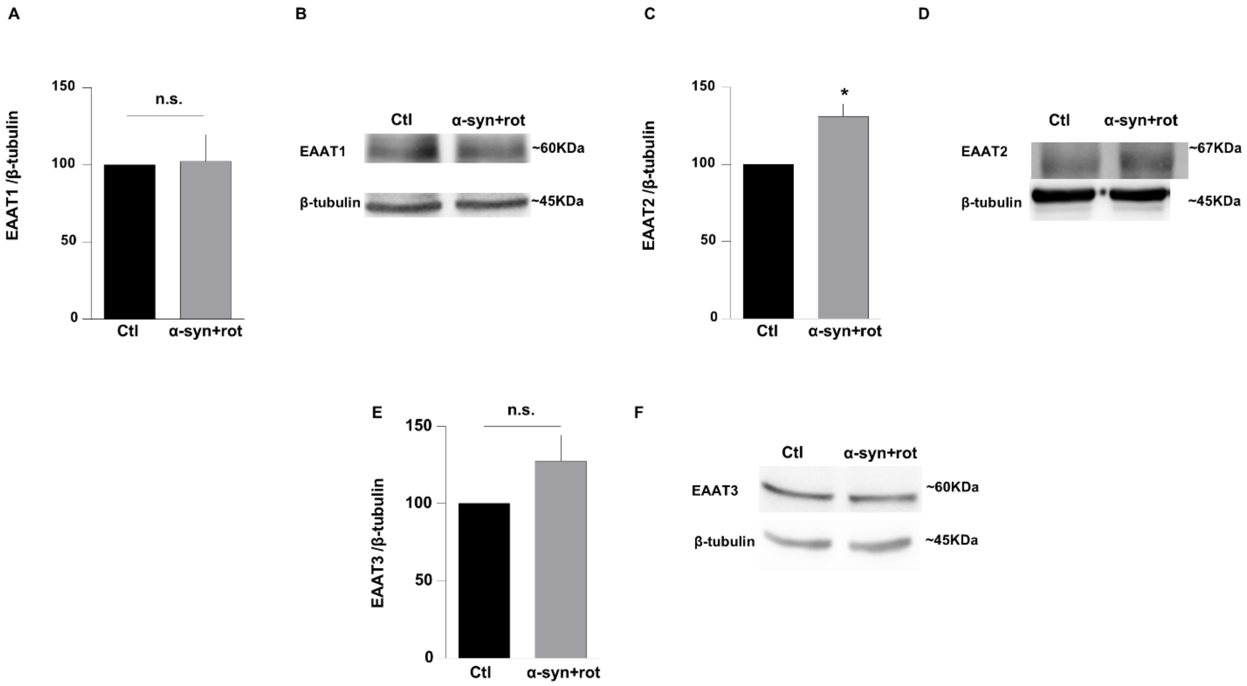

Figure S6. Effect of α-synuclein plus rotenone treatment on EAATs expression in primary rat mesencephalic neurons. A, C, E) Quantitative analysis of EAATs after α-syn + rot treatment in primary mesencephalic neurons. B, D, F) Representative western blot images of EAAT1 (GLAST is the rodent homologue) (B), EAAT2 (GLT-1 is the rodent homologue) (D) and EAAT3 (EAAC1 is the rodent homologue) (F) expression. Normalized optical density values are expressed as percentage of the respective control. Each column represents the mean ± S.E.M. of at least 3 independent experiments. Differences among means were assessed by Student's t-test. C) *Significant vs Ctl ($p < 0.05$).

Full western blot images

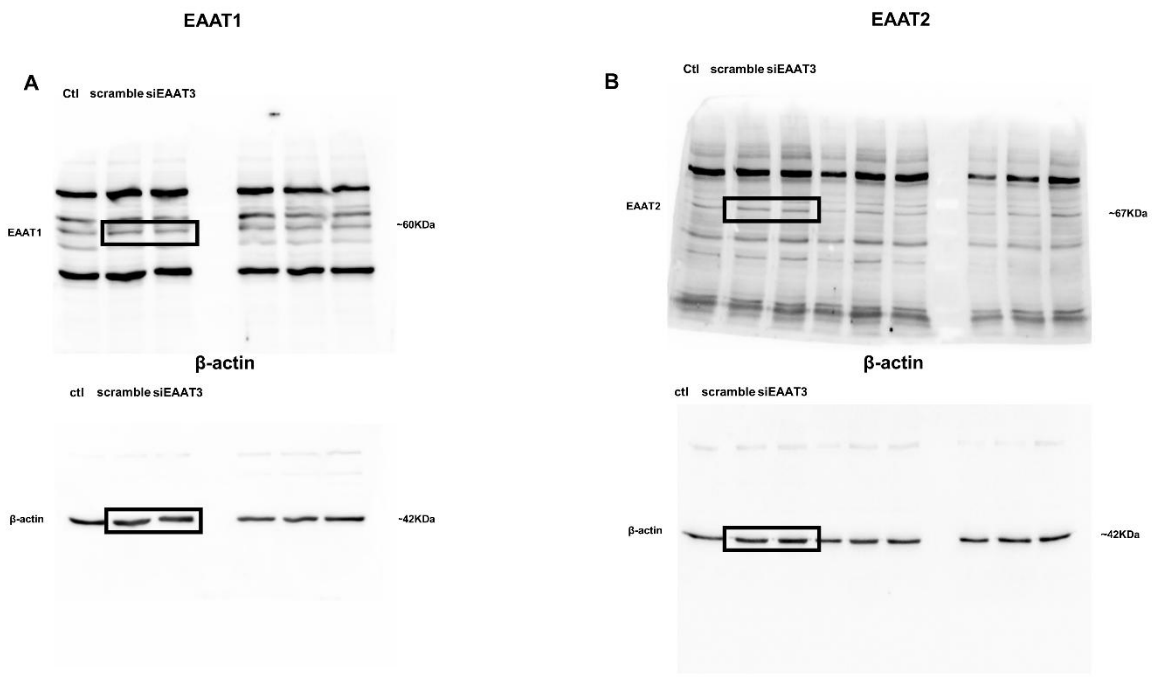
EAAT1
A
Ctl scramble siEAAT3
EAAT1
~60KDa
β-actin
ctl scramble siEAAT3
β-actin
~42KDa
EAAT2
B
Ctl scramble siEAAT3
EAAT2
~67KDa
β-actin
ctl scramble siEAAT3
β-actin
~42KDa

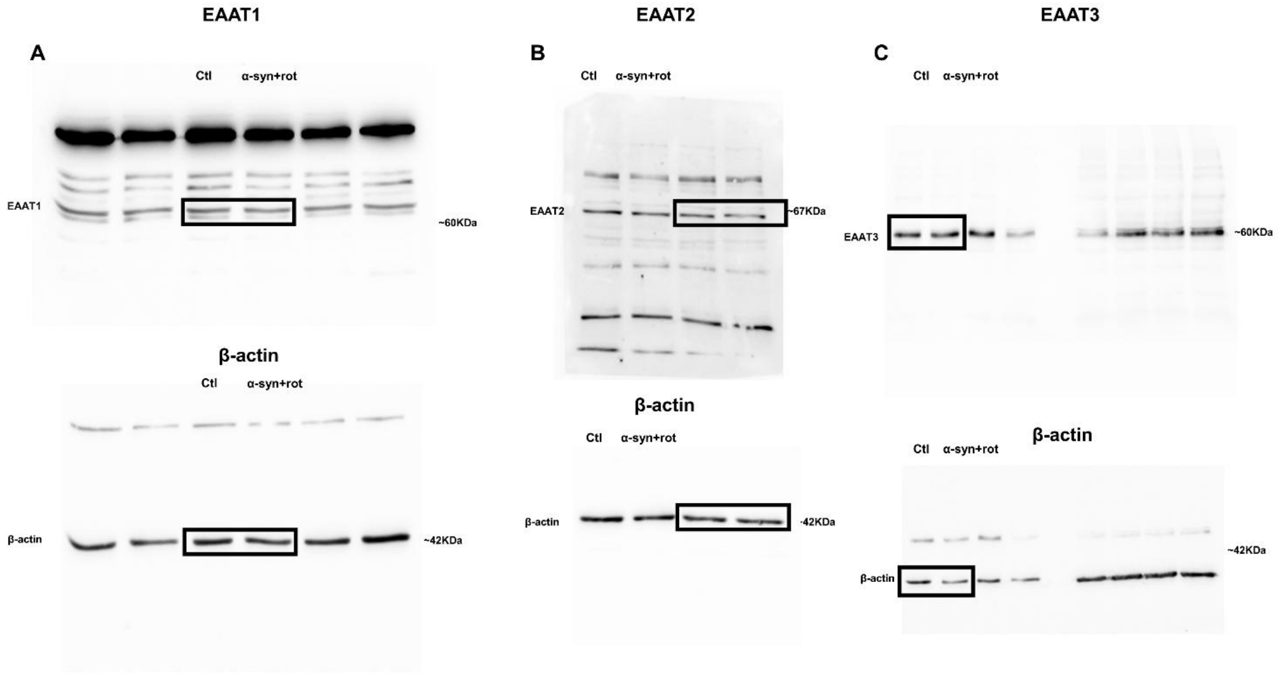
EAAT1
A
Ctl α-syn+rot
EAAT1
~60KDa
β-actin
Ctl α-syn+rot
β-actin
~42KDa
EAAT2
B
Ctl α-syn+rot
EAAT2
~67KDa
β-actin
Ctl α-syn+rot
β-actin
-42KDa
EAAT3
C
Ctl α-syn+rot
EAAT3
~60KDa
β-actin
Ctl α-syn+rot
~42KDa
β-actin

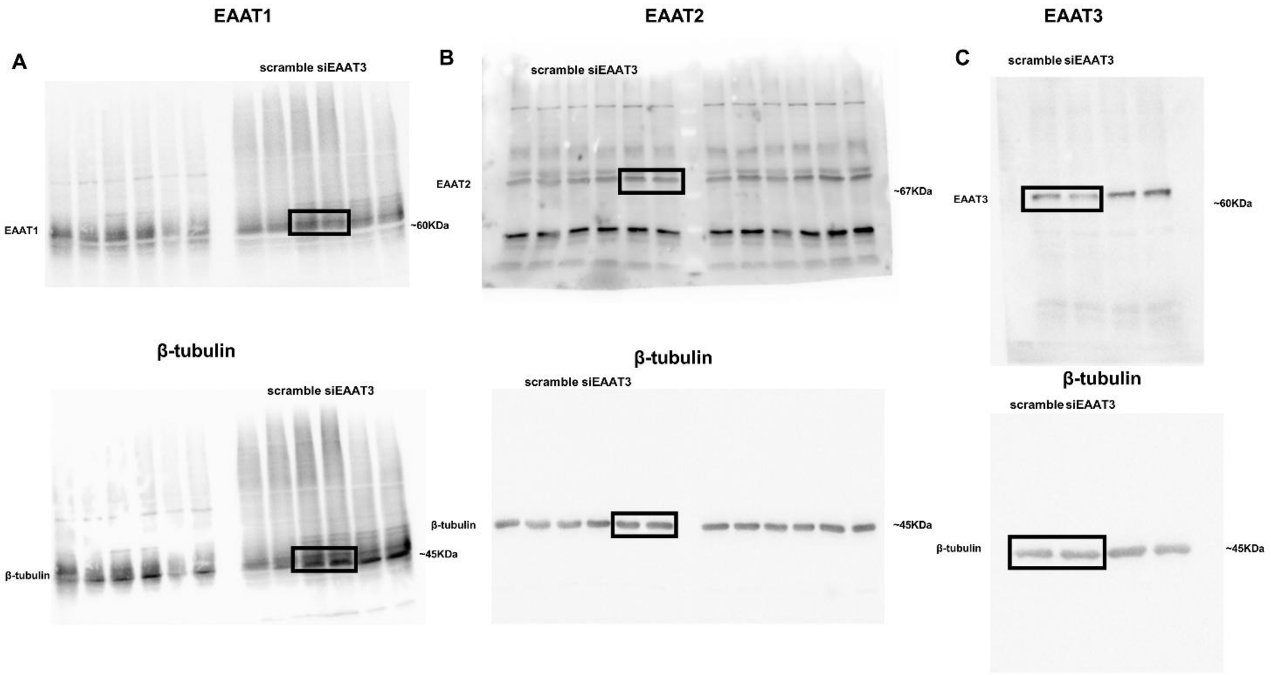
EAAT1
EAAT2
EAAT3
A
scramble siEAAT3
EAAT1
~60KDa
β-tubulin
scramble siEAAT3
β-tubulin
~45KDa
B
scramble siEAAT3
EAAT2
~67KDa
β-tubulin
scramble siEAAT3
β-tubulin
~45KDa
C
scramble siEAAT3
EAAT3
~60KDa
β-tubulin
scramble siEAAT3
β-tubulin
~45KDa

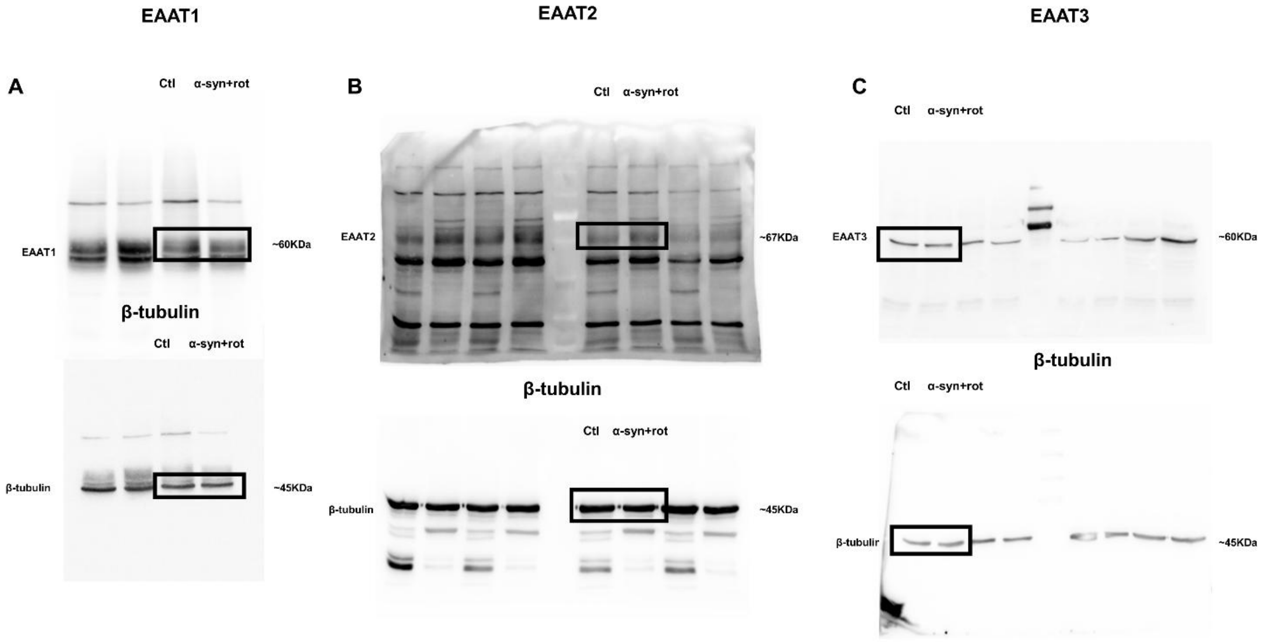
EAAT1
EAAT2
EAAT3
A
Ctl
α-syn+rot
EAAT1
~60KDa
β-tubulin
Ctl
α-syn+rot
β-tubulin
~45KDa
B
Ctl
α-syn+rot
EAAT2
~67KDa
β-tubulin
Ctl
α-syn+rot
β-tubulin
~45KDa
C
Ctl
α-syn+rot
EAAT3
~60KDa
β-tubulin
Ctl
α-syn+rot
β-tubulin
~45KDa
